# Computational Characterization of Undifferentially Expressed Genes with Altered Transcription Regulation in Lung Cancer

**DOI:** 10.3390/genes14122169

**Published:** 2023-12-01

**Authors:** Ruihao Xin, Qian Cheng, Xiaohang Chi, Xin Feng, Hang Zhang, Yueying Wang, Meiyu Duan, Tunyang Xie, Xiaonan Song, Qiong Yu, Yusi Fan, Lan Huang, Fengfeng Zhou

**Affiliations:** 1 Key Laboratory of Symbolic Computation and Knowledge Engineering of Ministry of Education, College of Computer Science and Technology, Jilin University, Changchun 130012, China; xrhcclg@163.com (R.X.); wyy180320@163.com (Y.W.); dmy235813@163.com (M.D.); huanglan@jlu.edu.cn (L.H.); 2 Jilin Institute of Chemical Technology, College of Information and Control Engineering, Jilin 132000, China; chengqian961025@163.com (Q.C.); xiaohangchi@gmail.com (X.C.); zhanghang2@jlict.edu.cn (H.Z.); 3 School of Science, Jilin Institute of Chemical Technology, Jilin 132000, China; fengxin@jlict.edu.cn; 4 Department of Epidemiology and Biostatistics, School of Public Health, Jilin University, Changchun 130012, China; yuqiong@jlu.edu.cn; 5 Centre for Mathematical Sciences, University of Cambridge, Wilberforce Road, Cambridge CB3 0WA, UK; xtygo0918@126.com; 6 Key Laboratory of Symbolic Computation and Knowledge Engineering of Ministry of Education, College of Software, Jilin University, Changchun 130012, China; songxn1009@163.com; 7 School of Biology and Engineering, Guizhou Medical University, Guiyang 550025, China

**Keywords:** bioinformatics, differential expression, transcription regulation, lung cancer, mqTrans, dark biomarker

## Abstract

A transcriptome profiles the expression levels of genes in cells and has accumulated a huge amount of public data. Most of the existing biomarker-related studies investigated the differential expression of individual transcriptomic features under the assumption of inter-feature independence. Many transcriptomic features without differential expression were ignored from the biomarker lists. This study proposed a computational analysis protocol (mqTrans) to analyze transcriptomes from the view of high-dimensional inter-feature correlations. The mqTrans protocol trained a regression model to predict the expression of an mRNA feature from those of the transcription factors (TFs). The difference between the predicted and real expression of an mRNA feature in a query sample was defined as the mqTrans feature. The new mqTrans view facilitated the detection of thirteen transcriptomic features with differentially expressed mqTrans features, but without differential expression in the original transcriptomic values in three independent datasets of lung cancer. These features were called dark biomarkers because they would have been ignored in a conventional differential analysis. The detailed discussion of one dark biomarker, GBP5, and additional validation experiments suggested that the overlapping long non-coding RNAs might have contributed to this interesting phenomenon. In summary, this study aimed to find undifferentially expressed genes with significantly changed mqTrans values in lung cancer. These genes were usually ignored in most biomarker detection studies of undifferential expression. However, their differentially expressed mqTrans values in three independent datasets suggested their strong associations with lung cancer.

## 1. Introduction

Lung cancer is one of the most invasive and lethal cancer types for both females and males [1,2]. More than 80% of lung cancers are non-small-cell lung cancers (NSCLC) [3], while the rest comprises small-cell lung cancer (SCLC) [4]. There are three subtypes of NSCLC [5], i.e., lung squamous-cell carcinoma (LUSC) [6], lung adenocarcinoma (LUAD) [7], and large-cell lung cancer [8]. Complicated molecular mechanisms are involved in the occurrence and progression of different lung cancer subtypes, and various diagnosis and treatment technologies have been developed based on these molecular mechanisms [9].

Biomedical imaging technologies undergo rapid developments to serve as a non-invasive diagnosis tool for malignant lesions like lung cancer. Mammography is a popular technology for detecting pulmonary nodules in lung cancer [10] and nodular breast lesions in breast cancer [11], but its usage in diagnosing lung cancer is limited [12]. Magnetic resonance imaging (MRI) uses a power magnetic field to create a 3D picture inside the body without radiations like X-rays, and it is very sensitive to the detection of internal inflammation lesions [13]. Low-dose computed tomography (LDCT) has been widely used in the diagnosis of lung cancer, especially in screening small peripheral pulmonary nodules and early-stage adenocarcinoma [14]. Positron emission tomography (PET) scans can reveal the metabolic status inside the body and are usually integrated with the computed tomography (CT) technology to create 3D PET/CT images of internal lesions [15].

A transcriptome is a set of the expression levels of the transcribable regions in the genome using microarray or sequencing technologies [16], and it is one of the most popular OMIC data types with abundant publicly available datasets [17]. The transcriptome has been extensively used in the biological investigation of lung cancers [2]. Transcription factors (TFs) bind to sequence-specific genomic regions and regulate the expression levels of various transcripts [18]. A precise investigation of how the TFs are involved in the onset and development of lung cancers facilitates the detection of candidate therapeutic targets and improves the prognosis of lung cancers. The transcription factor STAT3 (signal transducer and activator transcription 3) is kept constitutively expressed and could be related to the tumorigenesis of lung cancer [19]. Another transcription factor, Pokemon (a central regulator of an important tumor suppressor gene, ARF), is expressed in non-small-cell lung cancers (NSCLC) by acting on the upstream regions of multiple proto-oncogenes and tumor suppressor genes [20]. The expression level of the well-known prognostic biomarker PD-L1 shows a strong correlation with the survival of multifocal lung cancer patients [21].

Our hypothesis is that previous studies have ignored many undifferentially expressed genes, whose transcription regulation may be quantitatively altered in a phenotype. We use the recently developed algorithm mqTrans [22,23] to quantitatively measure the transcription regulation machinery in healthy samples. Then, we screen three lung cancer datasets for undifferentially expressed genes whose quantitative transcription regulation is significantly altered in lung cancer samples compared to those in the healthy controls. The inter-feature correlations of these genes show significant associations with lung cancer, but these genes do not show differential expression themselves in one or more datasets. We call these genes the dark biomarkers of lung cancers, because their mqTrans values are substantially changed between the lung cancer samples and the controls, although their original expression levels remain undifferentially expressed.

## 2. Materials and Methods

### 2.1. Summary of the Datasets

We collected three datasets—GSE33356/GSE18842/GSE30219—from the Gene Expression Omnibus (GEO) database [24] for screening and independently validating undifferentially expressed genes with altered transcription regulation in lung cancer. All the three datasets were transcriptomes profiled on the platform GPL570 [25]. There were 54,675 transcriptomic features per sample, and they were annotated as the mRNA gene features or transcription factor (TF) features based on the annotations of gene symbols in the platform annotation release 36 of the Human Genome U133 Plus 2.0 Array and the database Human TFDB [26]. Dataset GSE33356 consisted of 60 lung cancer samples and 60 healthy controls. There were 46 lung cancer and 45 control samples in dataset GSE18842. The third dataset, GSE30219, had 293 lung cancer samples and 14 controls. The datasets were retrieved from the GEO database after a standard preprocessing step [24].

We used 70% of the healthy control samples of dataset GSE33356 to train the mqTrans model and evaluated the mqTrans features on 100% of the lung cancer samples and the remaining 30% of the controls in the same dataset. The results were further screened with two independent datasets, GSE18842 and GSE30219. The detailed description of the experimental procedure is discussed in the following sections.

### 2.2. Expression Prediction Using Upstream TFs

Gene expression is strictly controlled by the transcription regulation machinery, and this study assumes that a gene’s expression level may be formulated as a regression model of the TFs’ expression levels.

Such a regression model is defined as a linear regression function [27] between the expression levels of one mRNA gene and multiple TFs, i.e., LinearR(*mRNA*) = *W*_0_ + *W*_1_ × *TF*_1_ + … + *W_n_* × *TF_n_*, where *W_i_* is the weight of *TF_i_*, and *W*_0_ is a constant. The algorithm is implemented using the LinearRegression function of the package sklearn.linear_model [28] in the Python programming language version 3.8.

The Pearson correlation coefficient (PCC) is used to ensure the quality of regression models. Let the predicted and real expression levels of a gene F be *mRNA’*(*F*) and *mRNA*(*F*). The gene is kept for further screening only if PCC(*mRNA’*(*F*), *mRNA*(*F*)) > 0.5. Another metric, the root mean square error (RMSE), has been also popularly used to measure the average difference between a model’s predicted and actual values. This study follows [29,30] in using the PCC to measure the correlation between the predicted values and the original expression levels of an mRNA gene.

### 2.3. Calculation of the mqTrans Features

This study hypothesized that the transcription regulation of some genes was quantitatively maintained among healthy control samples and that the altered transcription regulation of a gene could be quantitatively measured from the difference of this gene’s real expression level to the predicted level using a regression model trained on the healthy controls. Therefore, the difference between the predicted and real expression levels of a gene *F* is defined as the engineered mqTrans feature, as follows: *mqTrans*(*F*) = |*mRNA’*(*F*) − *mRNA*(*F*)|. The mqTrans model was trained using the healthy control samples, and the predicted expression level of a screened gene *F* was ensured to be highly correlated with the real level using a PCC(*mRNA’*(*F*), *mRNA*(*F*)) > 0.5. So, a gene *F*’s mqTrans feature, *mqTrans*(*F*), tends to be close to zero if this gene’s transcription regulation in the current query sample is quantitatively maintained in the same pattern as the training healthy samples [23].

This study engineered the mqTrans features of the testing samples for all the original features using the trained regression mqTrans models and the high correlations between the predicted and real expression levels in the training samples.

The differential expression was evaluated between the lung cancer (positive) and healthy control (negative) samples using the *p*-value of a *t*-test [31]. The statistical *t*-test was implemented using the function ttest_ind_from_stats of the package scipy.stats in the Python programming language version 3.8. A feature was supposed to be significantly associated with lung cancer if its *t*-test *p*-value < 0.05. The lung cancer and control samples were evaluated in both the original and mqTrans feature spaces.

### 2.4. Experimental Design

**Figure 1:**
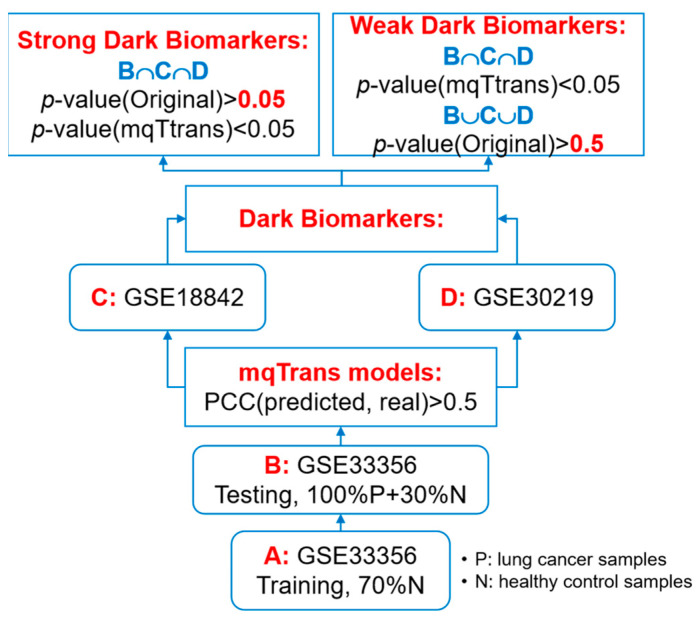
Flowchart of the experimental design of this study.

This study plans to screen for the transcriptomic features that are not differentially expressed in lung cancer samples but whose transcription regulation is significantly altered in lung cancer, as illustrated in Figure 1. We firstly train mqTrans models using 70% of the healthy controls in dataset GSE33356 and ensure the regression qualities with a requirement of PCC(*mRNA’*(*F*), *mRNA*(*F*)) > 0.5, where *mRNA’*(*F*) and *mRNA*(*F*) are the predicted and real expression levels of a gene *F*. Then, we calculate the mqTrans features for those transcriptomic features with the above-trained mqTrans models. 

We screen two groups of dark biomarkers, as shown in Figure 1. We define a transcriptomic feature as a dark biomarker between the disease group and the control group if its original values are not differentially expressed (*p*-value(Original) > 0.05) and its mqTrans values are differentially expressed (*p*-value(mqTrans) < 0.05). A strong dark biomarker meets the dark biomarker requirement in all the investigated datasets, while a weak dark biomarker only meets the requirement in one dataset and has a *p*-value(Original) > 0.5 in the same dataset.

## 3. Results and Discussion

### 3.1. Data Preprocessing

This study screened the dark biomarkers of lung cancer samples in three independent datasets (GSE33356, GSE18842, and GSE30219). The transcriptomic features without annotations in the “Gene Symbol” of the platform data of GPL570 were excluded. Among the remaining 45,782 features, 3501 features were annotated as the transcription factor (TF) features based on the information from the database Human TFDB [26]. The transcriptomic data were normalized to [0, 1] [32].

The above section, “Summary of the Datasets”, showed that dataset GSE33356 has the largest number of healthy control samples. So, 70% of the healthy controls in this dataset were randomly retrieved to train unsupervised mqTrans models. The remaining samples of dataset GSE33356 were used as the testing dataset. The other two datasets, GSE18842 and GSE30219, were used to independently test the detected biomarkers. 

### 3.2. The Quantitative Transcription Regulatory Models

Many OMIC data sources can be integrated to computationally calculate a gene’s expression [33]. Sequence features have been previously explored for their correlations with the gene expression levels in Caenorhabditis elegans via a Bayesian probabilistic framework [34]. TF-binding statuses with the ChIP-seq and chromatin data have been integrated in a regression model to predict gene expression [35]. Deep learning algorithms, like convolutional neural network (CNN), have been used to consolidate the cis signals in promoters and distal regulatory regions for the prediction of cell-type-specific gene expression [36]. But, the transcriptome is one of the OMIC types with the most abundant public datasets and open-source analysis tools [22,23,37].

**Figure 2:**
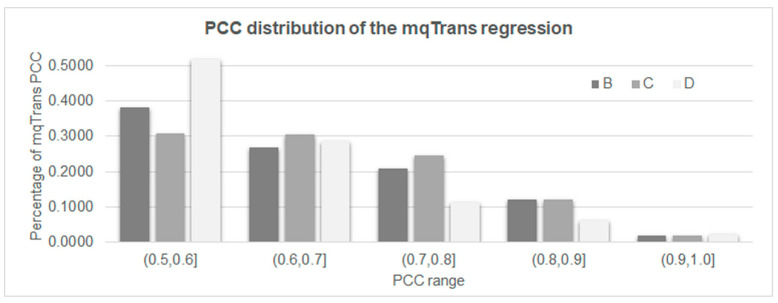
The distribution of the PCC values of the transcriptomic features with a PCC > 0.5 in all four datasets. Dataset A consists of the 70% randomly chosen healthy control samples from dataset GSE33356 for training the mqTrans models, and the remaining samples of dataset GSE33356 constitutes dataset B. Datasets C and D represent datasets GSE18842 and GSE30219.

We screened for mqTrans regression models trained in a dataset A with PCC > 0.5 between the predicted and real expression levels in a dataset B, as shown in Figure 2. A threshold PCC > 0.5 has been previously used to determine the co-expression patterns of two biological molecules [38]. There were 5820 mqTrans regression models achieving a PCC > 0.5 in dataset B. A total of 2396 and 1146 of these models kept achieving a PCC > 0.5 in the two datasets C and D, respectively. There were 116 mqTrans models achieving a PCC > 0.9 on the validating samples of the same dataset, GSE33356. Even on the two independent testing datasets C (GSE18842) and D (GSE30219), 49 and 24 mqTrans regression models achieved a PCC > 0.9 between the predicted and real expression levels.

### 3.3. Differential Transcription Regulation Analysis

The engineered mqTrans features were screened for their differential representations between the two groups of lung cancer and healthy control samples. The mqTrans feature of the original transcriptomic feature *F* in a query sample *S* was defined as *mqTrans*(*F*) = |*mRNA’*(*F*) − *mRNA*(*F*)|, where the transcriptomic value of *F* was *mRNA*(*F*), and its predicted expression was *mRNA’*(*F*). The expression prediction model was trained using the healthy controls. So, the engineered *mqTrans*(*F*) quantitatively measured the change of the *F*’s transcription regulation in the query sample *S* compared with the training group of the healthy control samples.

A differential transcription regulation (DeTouR) analysis was conducted to calculate the differential representations of the mqTrans features between the two groups of lung cancer and healthy control samples using the above formulations. The mqTrans regression models were trained using randomly extracted healthy controls, and the differential analysis used 0.05 as the significance threshold of the *t*-test *p*-values.

**Figure 3:**
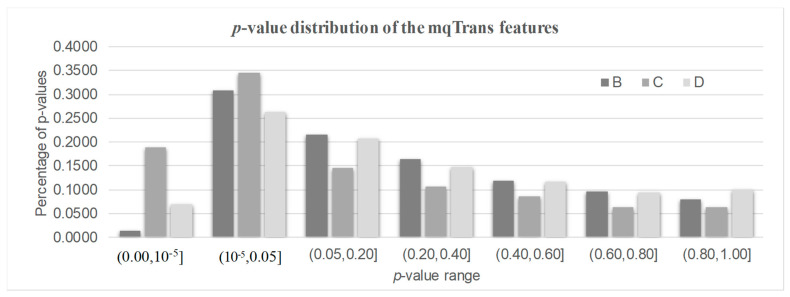
The distribution of the *p*-values of the mqTrans features in all three testing datasets. The 30% healthy controls and 100% lung cancer samples of dataset GSE33356 constitute dataset B. Datasets C and D represent datasets GSE18842 and GSE30219.

There were 1880, 1278, and 381 features with a significantly altered transcription regulation in the three datasets B/C/D, as shown in Figure 3. We called them the DeTouR features. There were about 1/3 of mqTrans features whose transcription regulation was significantly altered in the two datasets B (32.30%) and D (33.25%), respectively. More than half (53.34%) of the mqTrans features in dataset C were differentially transcriptionally regulated. There were even 80, 452, and 79 mqTrans features with DeTouR *p*-values < 0.05 in the three datasets B/C/D, respectively.

### 3.4. DeTouR Features Ignored by a Conventional Differential Analysis

We further screened the DeTouR features that would have been ignored in a conventional differential analysis. Most of the existing biomarker detection studies evaluate the differential significance of a given feature between two groups of samples using a statistical test like the *t*-test [39]. Many features with a statistical significance of *p*-values > 0.05 would be ignored, with the assumption that these features are not associated with the investigated phenotypes.

We focused on those DeTouR features whose original expression was not differentially expressed in the respective datasets, as shown in Table 1 and in Supplementary Table S1. The PCC values in Table 1 show that the expression levels of these DeTouR features were confidently predicted. Two DeTouR features, 208296_x_at and 229625_at, were detected to be differentially represented in the mqTrans level but not in the original expression level in all three datasets B, C, and D. The mqTrans features of these two features were differentially expressed in the lung cancer samples (*p*-values < 0.05), while their original expression levels were not (*p*-values > 0.05) in all three datasets B, C, and D. The original expression levels of 229625_at were almost identical between the two groups of lung cancer and healthy control samples in the two datasets GSE33356 (*p*-value = 7.42 × 10^−1^) and GSE30219 (*p*-value = 6.40 × 10^−1^). These two features were regarded as strong dark biomarkers from the mqTrans view of all three datasets B, C, and D, although they would have been ignored by a conventional differential expression analysis using any of these three datasets. If we had used a conventional *t*-test to rank these features, both of these two strong dark biomarkers would have ranked lower than 5000 in all three datasets, dbB/dbC/dbD (Supplementary Table S1). Most studies would not investigate such lowly ranked features.

We observed that some DeTouR features had very large *p*-values at their original expression levels. So, we included 11 additional DeTouR features as the weak dark biomarkers if their original expression levels had *p*-values > 0.5 in at least one dataset. For example, the original expressions of DeTouR feature 225107_at showed almost identical distributions between the lung cancer and healthy control samples, with a *p*-value = 9.54 × 10^−1^ in dataset B. Another DeTouR feature, 203954_x_at, showed highly similar distributions between the lung cancer and healthy control samples in the original expression levels in the two datasets C (*p*-value = 6.33 × 10^−1^) and D (*p*-value = 2.84 × 10^−1^). These 11 additional features could have been ignored in a conventional differential analysis, at least in some datasets, and were regarded as the weak dark biomarkers.

**Figure 4:**
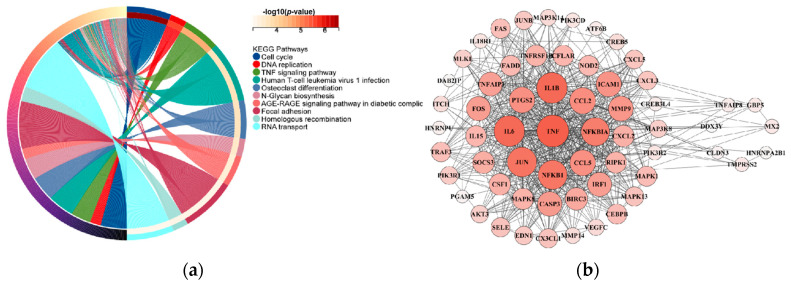
Lung cancer differentially expressed gene enrichment analysis and PPI network analysis of 13 dark biomarker genes. (**a**) The left side of the figure displays the top ten pathways from the KEGG enrichment analysis results. Specifically, we selected 54 genes in the TNF signaling pathway. (**b**) On the right side, the protein–protein interaction (PPI) analyses yield results indicating that seven dark biomarkers have node interactions with lung cancer significant difference genes.

We performed a functional enrichment analysis and a protein–protein interaction network assessment for the 13 dark biomarker genes associated with lung cancer. For the functional enrichment analysis, we used the KEGG rest API (https://www.kegg.jp/kegg/rest/keggapi.html, accessed on 1 November 2023) to obtain the latest gene annotations of the KEGG pathway as the background, mapped the dark biomarker genes to the background set, and performed an enrichment analysis using the R software package clusterProfiler (version 3.14.3) [40] to obtain the gene set enrichment results. The minimum gene set size was set to five, and the maximum gene set size was set to five thousand, with a *p* value < 0.05. Protein–protein interaction (PPI) analyses were conducted utilizing the online STRING database [41], which provides functional protein association networks (https://cn.string-db.org/, accessed on 1 November 2023). Additionally, the protein interaction mapping was performed using the Cytoscape local client (Cytoscape_v3.6.1). The results in Figure 4 show that there were ultimately seven dark biomarkers identified to interact with genes within the TNF signaling pathway, which is actively involved in the development and metastasis of lung cancer [42,43,44].

### 3.5. Differential Patterns in the Two Levels

**Figure 5:**
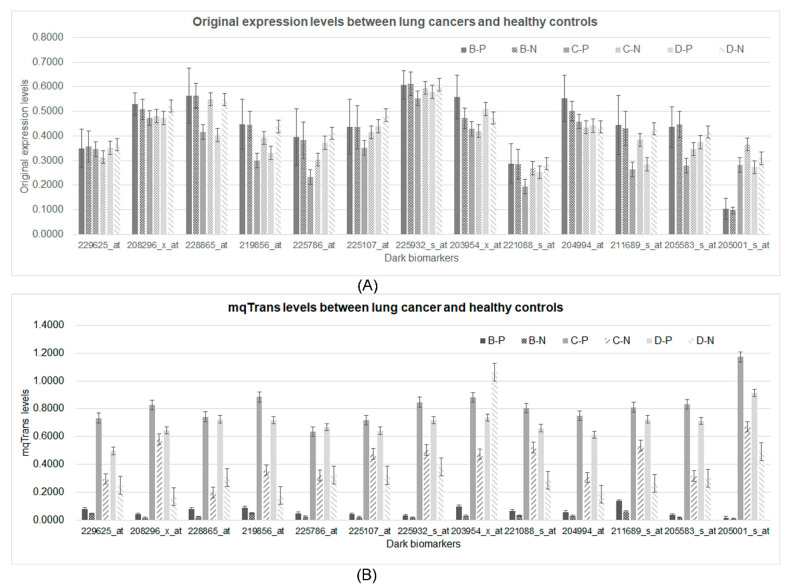
Comparison of the 13 dark biomarkers from both the original expression and mqTrans levels. (**A**) The original expression levels and (**B**) the mqTrans levels of these 13 dark biomarkers are displayed. The two strong dark biomarkers are on the left-most part. The horizontal axis lists the 13 dark biomarkers. The vertical axis illustrates the values of the respective feature levels. The data series B-P and B-N are the lung cancer and healthy control samples in dataset B. The other four data series, C-P, C-N, D-P, and D-N, are defined for the lung cancer and healthy control samples in datasets C and D, respectively.

**Figure 6:**
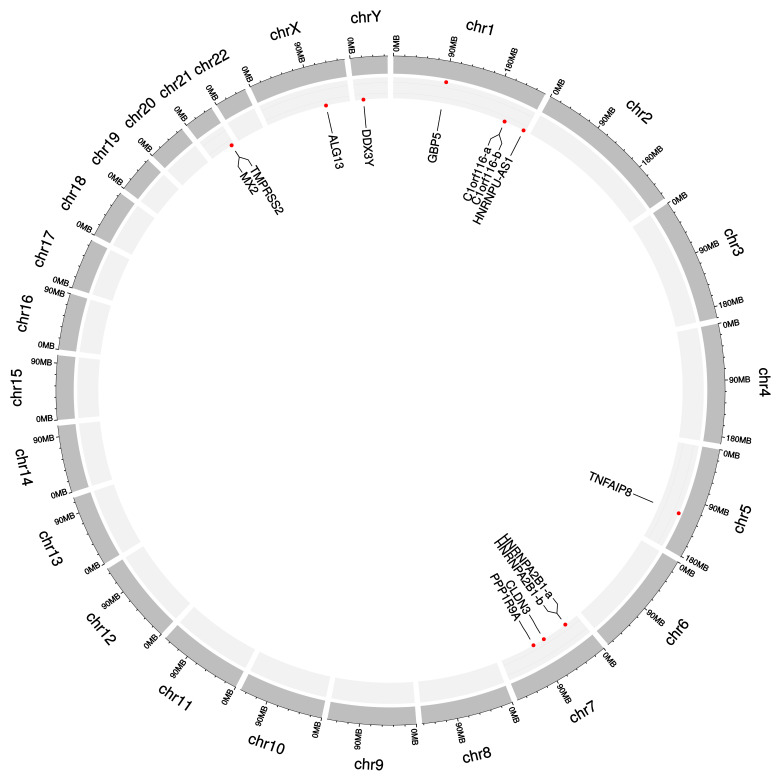
Circos plot of the 13 dark biomarkers in the human genome. The dark biomarkers are represented with the genes where they reside. The two strong dark biomarkers 229625_at (gene GBP5) and 208296_x_at (gene TNFAIP8) are highlighted in a larger size and red color. The two dark biomarkers 228865_at and 219856_at are both within the gene C1orf116, and they are denoted as C1orf116-a and C1orf116-b, respectively. Another pair of dark biomarkers, 225107_at and 225932_s_at, are from gene HNRNPA2B1, and they are denoted as HNRNPA2B1-a and HNRNPA2B1-b, respectively. The Circos plot was generated using the online version of shinyCircos.

The original expression levels of all 13 dark biomarkers showed limited differences between the lung cancer and healthy control samples, as shown in Figure 5A. If we took the ratio of the average values between the lung cancer and healthy control samples (P/N ratio) to measure the difference of a feature between these two sample groups, the original expression levels of these 13 features would have P/N ratios between [0.9752, 1.1824], [0.6864, 1.1033], and [0.6626, 1.0762] in the three datasets B, C, and D, respectively. These 13 features showed much larger changes in the engineered mqTrans levels than those in the original expression levels, illustrated via the distributions of the *t*-test *p*-values (Supplementary Figure S1). The minimum and maximum P/N ratios of these mqTrans features reached 1.6559 and 3.3574 in dataset B. Similar increased changes were observed in the other two datasets. We used the online tool shinyCircos to visualize the distribution of the thirteen dark biomarkers [45], and we observed that these dark biomarkers were distributed across six chromosomes (Figure 6). There were four dark biomarkers in each of the two chromosomes 1 and 7. The two strong dark biomarkers, GBP5 and TNFAIP8, were in chromosomes 1 and 5. 

A large-scale evaluation of the expression patterns of the two strong dark biomarkers GBP5 and TNFAIP8 across different human organs was conducted (Supplementary Figure S2). The GTEx Portal [46] visualized the gene expression profiles across most human organs, and Supplementary Figure S2a,b illustrates that these two dark biomarkers, GBP5 and TNFAIP8, showed relatively high expression levels in the lung compared to the other organs. We further investigated how these two genes were expressed in different cancer types compared against their matched normal samples (Supplementary Figure S2c,d). The visualizations were retrieved from the GEPIA database [47] using the TCGA data [48]. Both strong dark biomarkers had increased expression levels in tumor samples of many cancer types, including DLBC (lymphoid neoplasm diffuse large B-cell lymphoma), GBM (glioblastoma multiforme), OV (ovarian serous cystadenocarcinoma), PAAD (pancreatic adenocarcinoma), SARC (sarcoma), and TGCT (testicular germ cell tumors). But, GBP5 and TNFAIP8 were expressed at similar levels between the tumor and normal samples in the two lung cancer subtypes LUAD (lung adenocarcinoma) and LUSC (lung squamous-cell carcinoma), which confirmed our computational analysis results.

**Figure 7:**
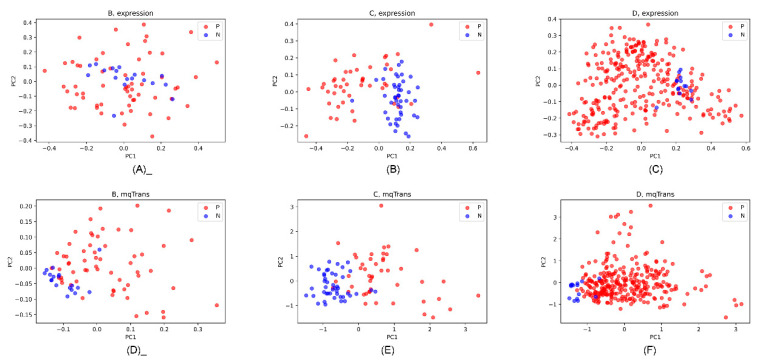
PCA dot plots of the 13 dark biomarkers on the original expression and mqTrans levels in the three datasets. The first and second principal components (PC1 and PC2) are used as the horizontal and vertical axis. The lung cancer and healthy control samples are colored as red (P) and blue (N). The PCA dot plots of the original expression levels are for datasets (**A**) B, (**B**) C, and (**C**) D. The PCA dot plots of the mqTrans levels are also generated for the three datasets (**D**) B, (**E**) C, and (**F**) D.

Figure 7 illustrates the dot plots of the lung cancer and healthy control samples on the original expression and mqTrans levels. The original expression levels of these 13 features did not separate the lung cancer samples from the healthy controls. But, the top two principal components [49] based on the 13 corresponding mqTrans features clearly separated the lung cancer samples from the healthy controls. Figure 7 indicates that useful information is carried by these dark biomarkers which might be ignored in a conventional differential analysis.

### 3.6. Validation of the Dark Biomarkers on an Independent Dataset

We conducted an additional validation experiment of the detected dark biomarkers in independent datasets. A comprehensive screening was carried out for the lung cancer datasets in the GEO database [24]. We found four new GPL570-based datasets with at least 100 samples, i.e., GSE115458, GSE18385, GSE33532, and GSE19188. Datasets GSE115458 and GSE18385 had no normal control samples. Dataset GSE33532 consisted of only twenty patients with diagnosed early-stage non-small-cell lung cancer (NSCLC), and each patient had four tumor sub-samples and one matched normal lung tissue sample. Therefore, we could only obtain one independent validation dataset, GSE19188.

The same mqTrans protocol confirmed the three dark biomarkers in Table 1 using the independent validation dataset GSE19188. The strong dark biomarker 229625_at (gene symbol: GBP5) showed a significant association (mqTrans-*p* = 3.54 × 10^−3^) with lung cancer in dataset GSE19188, while its original expression maintained very stable expression levels with a *p*-value = 0.9113 between the two groups of lung cancer and control samples. The weak dark biomarker 203954_x_at (gene symbol: CLDN3) also showed stable expression levels (*p*-value = 0.6916) between the lung cancer and control samples, while its mqTrans values were significantly associated with lung cancer (mqTrans-*p* = 4.34 × 10^−3^). The other weak dark biomarker 204994_at (gene symbol: MX2) had similar patterns in the dataset.

### 3.7. Biological Observation of the Strong Dark Biomarker GBP5

The strong dark biomarker GBP5 encodes the Guanylate-Binding Protein 5 [50] and is actively involved in the innate immune system and the interferon γ signaling pathway [51]. GBP5 was recently observed to be significantly differentially expressed in liver cells under different interferon γ treatments and may potentially facilitate the finding of effective treatment for the Hepatitis B Virus (HBV) [52]. Recent studies also found that GBP5, together with a few other genes, served as an ideal and stable diagnosis biomarker set for pulmonary tuberculosis (TB) [53]. 

Although no literature supported any connections between GBP5 and lung cancer, its co-transcribed paralog GBP1 was observed to be involved in lung adenocarcinoma (LUAD). Yamakita et al. experimentally demonstrated that GBP1 facilitated the metastasis process of LUAD and that its expression needed to be actively repressed to control the LUAD’s progression [54]. The resistance to the first-line erlotinib treatment of LUAD might be promoted by the up-regulated GBP1 expression [55]. The close correlation of GBP1 with advanced LUAD’s profiles was successfully utilized in the prognosis prediction and management of LUAD patients [55].

A curation of the long non-coding RNAs’ overlapping with the 13 dark biomarker features was collected from the LncBook database [56] (the phenotype “Lung cancer” in Supplementary Table S2). GBP5 has an antisense long non-coding RNA (lncRNA), HSALNG0005054, without detailed investigations in the literature. HSALNG0005054 was only transcribed in infant (after 18 weeks) and elderly livers and had no detectable expression in newborn or adolescent livers [57]. No expression was detected in the development of the other six organs. The data suggested that this lncRNA was under a precise transcription regulation of its functions.

This study analyzed three independent datasets to show that the expression levels of GBP5 did not show differential expression in lung cancer but that its quantitative correlations with its upstream TFs were significantly altered. Combined with the above observations in the literature, how GBP5 is involved in the onset of lung cancer might be worth further experimental investigations.

### 3.8. RNA-Seq Dark Biomarkers of Late-Stage LUAD and LUSC

We evaluated the proposed mqTrans protocol on the dark biomarkers of late-stage lung cancer using the RNA-seq transcriptomes from The Caner Genome Atlas (TCGA) database [48]. Due to the fact that the number of control samples is extremely small in the TCGA database, this section focused on the two classes of early- and late-stage lung cancer. The two subtypes lung adenocarcinoma (LUAD) and lung squamous-cell carcinoma (LUSC) of lung cancer were used for the evaluation. Stages I and II were denoted as early-stage lung cancer, and stages III and IV were the late-stage cancer samples. Only the samples with annotations of the stages I/II/III/IV were used for the analysis in this section. We randomly extracted 70% of the early-stage samples to train the regression models and conducted a Kaplan–Meier (KM) survival analysis based on the original expression and the mqTrans values of the detected dark biomarkers of each lung cancer subtype [58].

Supplementary Table S3 shows that fourteen and two dark biomarkers were detected for the late-stage LUAD and LUSC samples, respectively. Their overlapping lncRNAs are listed in the phenotypes “Late LUAD” and “Late LUSC” in Supplementary Table S2. The transcription regulation of these 16 dark biomarkers was significantly altered in the late-stage lung cancer samples, while their original expression levels remained unchanged compared to the early-stage samples. It is interesting to observe that the novel transcript ENSG00000267249 (gene symbol: RP11-973H7.3) maintained very stable expression levels (raw-*p* = 0.4012) between the early- and late-stage LUAD samples, while it showed significantly different mqTrans values (mqTrans-*p* = 8.28 × 10^−4^). ENSG00000267249 also showed very stable expression levels across different human organs, while it was highly expressed in the brain cerebellar hemisphere and the brain cerebellum (Supplementary Figure S3) based on the UCSC Genome Browser [59]. This gene had no orthologs across rats, zebrafish, and flies. So, ENSG00000267249 might be a good candidate for the investigation of how human-specific lung adenocarcinoma develops, since this gene has received very limited attention in the literature. 

**Figure 8:**
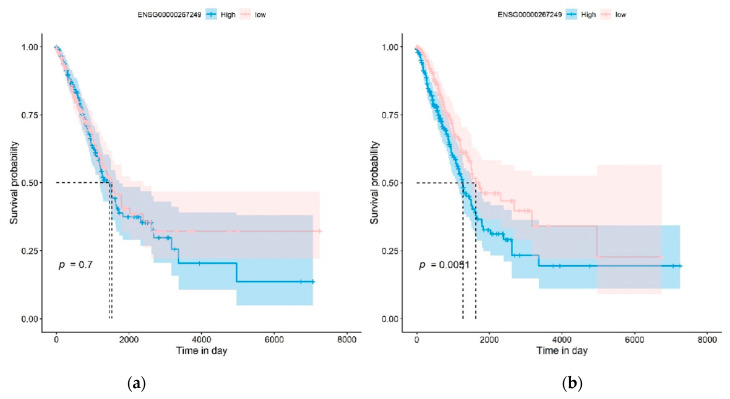
Kaplan–Meier (KM) survival analysis of the dark biomarker ENSG00000267249 (gene symbol: RP11-973H7.3) in the LUAD experiment. The KM plots of (**a**) the original expression levels and (**b**) the mqTrans values of this dark biomarker are generated for LUAD, respectively.

We further investigated how the mqTrans features could improve the Kaplan–Meier (KM) survival analysis of lung cancer compared to the original expression levels of these dark biomarkers, as shown in Figure 8 and in Supplementary Figure S4. The KM analysis excluded the samples without death time and with missing values in these dark biomarker genes. We divided all the samples into high-risk or low-risk groups using the same method as in [58]. The KM survival analysis was conducted on the original expression levels and the mqTrans values of each dark biomarker gene. Figure 8 illustrates that the original expression levels of the novel transcript ENSG00000267249 had no capability in discriminating the high-risk and low-risk groups of LUAD patients (*p* = 0.7000), while its mqTrans values showed a much improved statistical significance (*p* = 0.0051) in discriminating the high-risk LUAD patients from the low-risk group. The statistical significance of the KM plot of this gene was on the same level as in [58]. Similar patterns may be found in the KM analysis of all the 16 detected dark biomarkers of lung cancer in Supplementary Figure S4.

### 3.9. Overlapping lncRNAs Could Be a Disturbing Factor

Many genes overlapped with lncRNAs, and the transcripts of both an mRNA gene and its overlapping lncRNAs could not be easily discriminated. We collected the known lncRNAs overlapping with the detected dark biomarkers in the three experiments from the LncBook database [56] (Supplementary Table S2). We can see that most of the detected dark biomarkers have overlapping lncRNAs. 

We took the gene STIM2 (Stromal Interaction Molecule 2) as an example. STIM2 was a dark biomarker associated with late-stage LUAD and overlapped with one sense and three antisense lncRNAs, as shown in Supplementary Table S2. The LncBook database proposed a novel approach to calculate the expression levels of some lncRNAs whose transcripts were discriminable from the overlapping genes [56]. The lncRNA HSALNG0033503 resided completely within the region of STIM2, and it had medium expression levels in 42.14% of the 337 biological conditions in the LncBook database. The three antisense lncRNAs, HSALNG0033504, HSALNG0033505, and HSALNG0033510, also showed a recognizable expression in many biological conditions. Considering the technical limitations of many existing expression calculation approaches, we proposed that the lncRNAs overlapping with an mRNA gene might have contributed transcripts disturbing the precise determination of this gene’s expression level.

## 4. Conclusions

This study proposed a computational protocol for analyzing transcriptomes from the view of high-dimensional inter-feature correlations. The mqTrans regression models employed also ensured the explainability of the engineered features. The demonstrative experiment detected two strong dark biomarkers and eleven additional weak dark biomarkers of lung cancer. These dark biomarkers showed no differential expression in at least one of the three independent datasets and, therefore, could be ignored in a conventional differential expression analysis. But, all these 13 dark biomarkers showed a significantly differential expression from the view of the mqTrans features.

We recognize the possibility of new transcription factors involved in the regulation of gene expression in a disease state. The models we have built are specific sets of transcription factors obtained with learning in healthy samples and used to predict the expression of the corresponding mRNA genes in a disease state. If new transcription factors emerge in the disease state and our model does not integrate these new factors, the prediction will be highly biased. We believe is the following to be one of the important contributions of our study: although the original expression of an mRNA gene may maintain similar values across diseased and healthy samples via different TF combinations, the mqTrans values are calculated based on the reference transcriptional regulatory network trained on the healthy samples and will change substantially in the disease samples, with new TFs for the corresponding mRNA genes.

A dark biomarker was not differentially expressed between the two groups of lung cancer and control samples, while the quantitative measurement of its transcription regulation showed a statistically significant differential expression in the lung cancer samples compared to the control group. Our detailed discussion of the strong dark biomarker GBP5 suggested that the overlapping lncRNAs might have contributed to this interesting phenomenon. Supplementary Table S2 provides additional evidence that many dark biomarkers have overlapping lncRNAs on both sense and antisense strands. Due to the inherent nature of microarray- and RNA-seq-based transcriptome profiling technologies, it is difficult to determine whether a detected transcript came from the mRNA or the lncRNA residing in the overlapping region. Therefore, the undifferential expression of an mRNA might consist of the transcripts of both the mRNA and the lncRNAs overlapping in the same region. Our mqTrans protocol provided a complementary way to detect these dark biomarkers that would otherwise be ignored in a conventional differential expression analysis.

We extend our analysis to single-cell RNA-seq (scRNA) datasets. The scRNA technology has recently emerged as a popular transcriptomic view to investigate the phenotypes of microbes, plants, and animals [60]. Two subsets of the GSE190725 study were used to compare the mqTrans analyses of both the bulk and single-cell RNA-seq data, i.e., endocrine cells and endocrine progenitor cells [61]. Supplementary Table S4 shows that the scRNA dark biomarkers are slightly fewer than the bulk RNA-seq dark biomarkers. Additionally, there are only four and seven dark biomarker genes supported by both the single-cell and bulk RNA-seq data for the endocrine cells and the endocrine progenitor cells, respectively. This suggests that an mqTrans analysis does not work well on scRNA data and may need further tuning to account for the characteristics of single-cell data.

The detection of dark biomarkers serves as an important and complementary analysis to the traditional differential expression analysis of transcriptomic biomarkers. Firstly, a traditional differential expression analysis detected 12593 biomarkers (*p*-values < 0.05) from the three datasets B/C/D and ignored 72.49% of the transcriptomic features. The mqTrans analysis can detect dark biomarkers with differential representations of lung cancer from the features ignored in a traditional biomarker analysis. Secondly, the mqTrans analysis provides supporting evidence for the protein-level phenotype associations of dark biomarkers without differential expression in wet-lab studies. The YTH N6-Methyladenosine RNA-binding protein C2 (YTHDC2) is an RNA-modification 6-methyladenine (m6A) reader [62] and was detected as the dark biomarker gene of metastatic colon cancer (mCC) [63]. Liu et al. observed that multiple m6A RNA methylation regulators showed differential expression in cancers except for YTHDC2 [64], and Tanabe et al. filled the gap with immunohistochemistry technology, showing that YTHDC2 is positively correlated with mCC on its protein levels [65]. Thirdly, some dark biomarkers show comparable expression levels to traditional biomarkers and merit further wet-lab investigations. Yoshimura et al. identified CD200 and CD200R1 as the differentially expressed biomarkers of lung cancer [66], while Supplementary Figure S5 shows that the two strong dark biomarkers have similar or higher expression levels compared to CD200 and CD200R1.

The proposed mqTrans protocol has a number of limitations to be resolved in future studies. Firstly, the computational analysis showed the altered transcription regulation of the dark biomarkers, and in vitro or in vivo investigations could be worth conducting on the possible interference of the expression of the long non-coding RNAs overlapping with these dark biomarkers. Secondly, regression was the main module of the mqTrans protocol and may be improved with feature selection algorithms and deep learning algorithms. 

In future studies, we will consider how to further refine the model to include potential new transcription factors and continuously improve the accuracy and robustness of the predictions. Different metrics like PCC and RMSE will also be evaluated to determine the measurement between the predicted values and the original levels of a given mRNA gene. The mqTrans analysis of various transcriptionally regulated targets like mRNA, lncRNA, and microRNA (miRNA) in different cancer types and other diseases remains largely unexplored and to be validated with wet-lab evidence.

## Figures and Tables

**Table 1:** Two types of dark biomarkers were detected in the three lung cancer datasets. There are “Strong” and “Weak” dark biomarkers indicated in the column “Type”. The next two columns, “Feature” and “Gene”, give the transcriptomic ID and gene symbol of the detected dark biomarkers. The three columns dbB/dbC/dbD indicate whether the feature satisfy the definition of a dark biomarker in datasets B/C/D, respectively. If the feature is a dark biomarker in a dataset, the corresponding column gives a value of 1. The three columns PCC-B/PCC-C/PCC-D indicate the PCC values in datasets B/C/D, respectively.

Type	Feature	Gene	dbB	PCC-B	dbC	PCC-C	dbD	PCC-D
Strong	229625_at	GBP5	1	0.5057	1	0.7275	1	0.8095
Strong	208296_x_at	TNFAIP8	1	0.5395	1	0.6465	1	0.6792
Weak	228865_at	C1orf116	1	0.5920	0	0.7263	0	0.5173
Weak	219856_at	C1orf116	1	0.5043	0	0.5716	0	0.5186
Weak	225786_at	HNRNPU-AS1	1	0.8405	0	0.7996	1	0.6197
Weak	225107_at	HNRNPA2B1	1	0.9013	0	0.7447	0	0.7005
Weak	225932_s_at	HNRNPA2B1	1	0.7947	0	0.6915	0	0.6522
Weak	203954_x_at	CLDN3	0	0.5682	1	0.5222	1	0.5282
Weak	221088_s_at	PPP1R9A	1	0.5592	0	0.5708	1	0.6123
Weak	204994_at	MX2	0	0.6886	1	0.6834	1	0.6351
Weak	211689_s_at	TMPRSS2	1	0.5350	0	0.5364	0	0.5124
Weak	205583_s_at	ALG13	1	0.8589	0	0.7153	0	0.5303
Weak	205001_s_at	DDX3Y	1	0.9180	0	0.7524	1	0.7513

## Data Availability

The source code and the example dataset were released on FigShare as https://doi.org/10.6084/m9.figshare.19614699.v3 (accessed on 1 November 2023).

## Supplementary Materials

The following supporting information can be downloaded at https://www.mdpi.com/article/10.3390/genes14122169/s1: Figures S1–S5 and Tables S1–S4.
