# Supplementary material for: Computational Characterization of Undifferentially Expressed Genes with Altered Transcription Regulation in Lung Cancer
Source: Genes (Basel). 2023 Dec 1;14(12):2169. doi: 10.3390/genes14122169 (PMC10742656; doi:10.3390/genes14122169)
Supplement: Supplementary file 1 [file genes-14-02169-s001.zip › genes-2668545-supplementary.pdf]

Computational characterization of undifferentially expressed genes with altered transcription regulations in lung cancers

Ruihao Xin[1,2], Qian Cheng[2,#], Xiaohang Chi[2,#], Xin Feng[4,3], Hang Zhang[2], Yueying Wang[1], Meiyu Duan[1], Tunyang Xie[5], Xiaonan Song[6], Qiong Yu[4], Yusi Fan[6,*], Lan Huang[1], Fengfeng Zhou[1,7,*].

1 College of Computer Science and Technology, and Key Laboratory of Symbolic Computation and Knowledge Engineering of Ministry of Education, Jilin University, Changchun, Jilin, China, 130012.
2 College of Information and Control Engineering, Jilin Institute of Chemical Technology, Jilin, Jilin, China,132000.
3 School of Science, Jilin Institute of Chemical Technology, Jilin, Jilin, China,132000.
4 Department of Epidemiology and Biostatistics, School of Public Health, Jilin University, Changchun, China, 130012.
5 Centre for Mathematical Sciences, University of Cambridge, Wilberforce Road, Cambridge CB3 0WA, UK
6 College of Software, and Key Laboratory of Symbolic Computation and Knowledge Engineering of Ministry of Education, Jilin University, Changchun, Jilin, China, 130012.
7 School of Biology and Engineering, Guizhou Medical University, Guiyang 550025, Guizhou, China.
# These authors contributed equally to this study.
* Correspondence may be addressed to Fengfeng Zhou: ffzhou@jlu.edu.cn . Lab web site: http://www.healthinformaticslab.org/ . Phone: +86-431-8516-6024. Fax: +86-431-8516-6024. Correspondence may also be addressed to Yusi Fan: fan_yusi@163.com .

Supplementary Table S1

Detailed information of the 13 dark biomarkers in the three datasets. The first three columns "Type", "Feature" and "Gene" gave the types, transcriptome feature IDs and gene symbols of these 13 dark biomarkers. In each of the three datasets dbB/dbC/dbD, the columns "Flag", "mqTrans-p", "raw-p" and "raw-rank" were whether this gene was the dark biomarker in this dataset, t-test p-value of the mqTrans values, t-test p-value of the original expression levels, and its rank based on the ascending order of the t-test p-value among all the 45,782 features.

Type	Feature	Gene	dbB				dbC				dbD			
			Flag	mqTrans-p	raw-p	raw-rank	Flag	mqTrans-p	raw-p	raw-rank	Flag	mqTrans-p	raw-p	raw-rank
Strong	229625_at	GBP5	1	2.11E-02	7.42E-01	5709	1	3.87E-06	5.70E-02	17278	1	1.97E-02	6.40E-01	5839
Strong	208296_x_at	TNFAIP8	1	3.90E-03	6.84E-02	28355	1	3.86E-02	4.19E-01	7383	1	3.80E-03	5.13E-02	24170
Weak	228865_at	C1orf116	1	8.60E-03	9.79E-01	482	0	1.30E-07	6.00E-08	36844	0	7.10E-03	1.00E-04	38132
Weak	219856_at	C1orf116	1	2.47E-02	9.28E-01	1606	0	6.70E-06	5.69E-06	33567	0	3.00E-04	7.00E-04	35603
Weak	225786_at	HNRNPU-AS1	1	4.40E-02	6.78E-01	7127	0	8.41E-05	3.88E-05	31708	1	2.17E-02	9.56E-02	21028
Weak	225107_at	HNRNPA2B1	1	2.00E-02	9.54E-01	1030	0	4.95E-03	3.42E-05	31840	0	1.38E-02	1.49E-02	28951
Weak	225932_s_at	HNRNPA2B1	1	1.00E-02	7.14E-01	6339	0	1.91E-03	3.70E-07	35676	0	2.34E-02	1.56E-02	28736
Weak	203954_x_at	CLDN3	0	1.00E-04	2.00E-04	41536	1	4.04E-04	6.33E-01	4349	1	9.00E-03	2.84E-01	13581
Weak	221088_s_at	PPP1R9A	1	5.40E-03	8.36E-01	3616	0	4.85E-03	9.00E-08	36624	1	3.00E-03	1.67E-01	17606
Weak	204994_at	MX2	0	1.11E-02	2.90E-02	32185	1	3.21E-05	1.63E-01	12822	1	5.00E-03	8.38E-01	2545
Weak	211689_s_at	TMPRSS2	1	3.00E-03	6.52E-01	7714	0	1.85E-02	1.10E-07	36483	0	2.30E-03	1.00E-04	37759
Weak	205583_s_at	ALG13	1	2.66E-02	6.00E-01	8963	0	2.10E-07	3.50E-07	35724	0	5.40E-03	1.06E-02	29881
Weak	205001_s_at	DDX3Y	1	3.66E-02	5.46E-01	10262	0	5.21E-04	4.92E-06	33710	1	3.94E-02	1.31E-01	19219

Supplementary Table S2

The lncRNAs overlapping with the 11 dark biomarker genes of lung cancers detected in the three datasets and the 5 dark biomarker genes detected in the TCGA datasets. The first column "Gene" was the gene symbol of the dark biomarkers. Some dark biomarker features were the probeset IDs and shared the same gene symbol. So there were 11 dark biomarker genes. The next five columns "lncRNA", "Chr", "Start", "End" and "S" were the ID, chromosome, start position, end position and strand of the overlapping lncRNA. The column "DB" was the data source where this lncRNA was annotated. The last column "Type" showed whether the lncRNA resided on the same ("sense") or opposite ("antisense") strand of the gene. The last column "Phenotype" denoted the three experiments investigated in this study, i.e., lung cancers versus control samples (Lung cancer), late- versus early-stage LUAD (Late LUAD) and late- versus early-stage LUSC (Late LUSC).

Gene	lncRNA	Chr	Start	End	S	DB	Type	Phenotype
CLDN3	HSALNG0058863	chr7	73734949	73775454	+	LncBook	antisense	Lung cancer
CLDN3	HSALNG0058869	chr7	73760753	73784181	-	LncBook	sense	Lung cancer
MX2	HSALNG0147191	chr21	41357143	41369978	-	RefLnc	antisense	Lung cancer
MX2	HSALNG0133203	chr21	41362021	41367306	+	LncBook	sense	Lung cancer
MX2	HSALNG0133206	chr21	41381751	41382231	+	LncBook	sense	Lung cancer
MX2	HSALNG0133207	chr21	41409317	41420581	-	LncBook	antisense	Lung cancer
DDX3Y	HSALNG0141213	chrY	12904849	12912525	+	LncBook	sense	Lung cancer
ALG13	HSALNG0139549	chrX	111671924	111679567	+	LncBook	sense	Lung cancer
ALG13	HSALNG0151248	chrX	111706649	111711101	-	HAVANA	antisense	Lung cancer
ALG13	HSALNG0139553	chrX	111710208	111710408	+	LncBook	sense	Lung cancer
ALG13	HSALNG0139555	chrX	111745586	111747473	+	LncBook	sense	Lung cancer
ALG13	HSALNG0139556	chrX	111760568	111766891	-	LncBook	antisense	Lung cancer

Gene	lncRNA	Chr	Start	End	S	DB	Type	Phenotype
ALG13	HSALNG0139557	chrX	111763569	111772741	+	LncBook	sense	Lung cancer
TNFAIP8	HSALNG0044395	chr5	119249045	119279096	-	LncBook	antisense	Lung cancer
TNFAIP8	HSALNG0044398	chr5	119341576	119344829	+	LncBook	sense	Lung cancer
TNFAIP8	HSALNG0044399	chr5	119341662	119355805	-	LncBook	antisense	Lung cancer
TNFAIP8	HSALNG0148957	chr5	119398815	119399298	-	FANTOM	antisense	Lung cancer
TMPRSS2	HSALNG0133209	chr21	41441056	41481066	-	HAVANA	sense	Lung cancer
TMPRSS2	HSALNG0133210	chr21	41463611	41464310	+	LncBook	antisense	Lung cancer
TMPRSS2	HSALNG0133211	chr21	41476530	41477546	+	LncBook	antisense	Lung cancer
TMPRSS2	HSALNG0133212	chr21	41507464	41525499	+	LncBook	antisense	Lung cancer
TMPRSS2	HSALNG0133213	chr21	41520532	41536310	-	LncBook	sense	Lung cancer
C1orf116	HSALNG0010067	chr1	207014830	207018900	+	LncBook	antisense	Lung cancer
C1orf116	HSALNG0010068	chr1	207023019	207024975	-	LncBook	sense	Lung cancer
PPP1R9A	HSALNG0059658	chr7	94832135	95214946	-	LncBook	antisense	Lung cancer
PPP1R9A	HSALNG0150006	chr7	94925620	94928614	-	StringTie	antisense	Lung cancer
PPP1R9A	HSALNG0059659	chr7	94998828	94999028	+	LncBook	sense	Lung cancer
PPP1R9A	HSALNG0059661	chr7	95139240	95140188	-	LncBook	antisense	Lung cancer
PPP1R9A	HSALNG0059662	chr7	95204564	95205044	+	LncBook	sense	Lung cancer
PPP1R9A	HSALNG0059663	chr7	95249844	95250053	-	LncBook	antisense	Lung cancer
PPP1R9A	HSALNG0059664	chr7	95267877	95268965	-	LncBook	antisense	Lung cancer
HNRNPA2B1	HSALNG0056804	chr7	26171511	26185286	-	RefSeq	sense	Lung cancer
HNRNPA2B1	HSALNG0056805	chr7	26190128	26193374	-	LncBook	sense	Lung cancer
HNRNPU-AS1	HSALNG0012112	chr1	244840243	244848912	-	LncBook	sense	Lung cancer
HNRNPU-AS1	HSALNG0012111	chr1	244842636	244859006	+	LncBook	antisense	Lung cancer

Gene	lncRNA	Chr	Start	End	S	DB	Type	Phenotype
GBP5	HSALNG0005054	chr1	89260582	89269754	+	LncBook	antisense	Lung cancer
RP11-973H7.3	HSALNG0120116	chr18	12670426	12671145	-	LncBook	sense	Late LUAD
ACP6	HSALNG0006818	chr1	147628660	147637378	+	LncBook	antisense	Late LUAD
ACP6	HSALNG0006821	chr1	147642250	147647375	+	LncBook	antisense	Late LUAD
ZFYVE26	HSALNG0102062	chr14	67739105	67740073	+	LncBook	antisense	Late LUAD
ZFYVE26	HSALNG0102064	chr14	67759709	67760017	-	LncBook	sense	Late LUAD
FN3KRP	HSALNG0119469	chr17	82717589	82718358	+	LncBook	sense	Late LUAD
FN3KRP	HSALNG0119471	chr17	82728058	82734894	-	LncBook	antisense	Late LUAD
FN3KRP	HSALNG0145189	chr17	82728916	82732815	+	RefSeq	sense	Late LUAD
C7orf60	HSALNG0060612	chr7	112522268	112896625	+	RefLnc	antisense	Late LUAD
C7orf60	HSALNG0060627	chr7	112903961	112905818	-	LncBook	sense	Late LUAD
C7orf60	HSALNG0060628	chr7	112908741	112914098	+	LncBook	antisense	Late LUAD
C7orf60	HSALNG0060629	chr7	112915135	112915424	-	LncBook	sense	Late LUAD
KIFAP3	HSALNG0008363	chr1	170021458	170022249	-	LncBook	sense	Late LUAD
KIFAP3	HSALNG0008364	chr1	170024077	170024684	+	LncBook	antisense	Late LUAD
KIFAP3	HSALNG0141960	chr1	170041476	170046229	+	RefSeq	antisense	Late LUAD
PLPPR2	HSALNG0124019	chr19	11355699	11356673	+	LncBook	sense	Late LUAD
PLPPR2	HSALNG0145851	chr19	11363943	11364491	-	FANTOM	antisense	Late LUAD
MECR	HSALNG0141714	chr1	29149139	29181819	+	FANTOM	antisense	Late LUAD
MECR	HSALNG0002110	chr1	29178734	29179198	-	LncBook	sense	Late LUAD
MECR	HSALNG0002111	chr1	29180743	29181058	-	LncBook	sense	Late LUAD
MECR	HSALNG0002112	chr1	29185581	29189710	-	LncBook	sense	Late LUAD
MECR	HSALNG0002113	chr1	29194795	29195790	-	LncBook	sense	Late LUAD

Gene	lncRNA	Chr	Start	End	S	DB	Type	Phenotype
MECR	HSALNG0002114	chr1	29196158	29201447	+	LncBook	antisense	Late LUAD
MECR	HSALNG0002115	chr1	29202207	29222701	-	LncBook	sense	Late LUAD
MECR	HSALNG0002116	chr1	29223267	29224817	+	Gnomon	antisense	Late LUAD
BPTF	HSALNG0118319	chr17	67802267	67825516	-	StringTie	antisense	Late LUAD
BPTF	HSALNG0118321	chr17	67826475	67827170	+	LncBook	sense	Late LUAD
BPTF	HSALNG0145355	chr17	67853935	67854767	-	FANTOM	antisense	Late LUAD
BPTF	HSALNG0118322	chr17	67875779	67885428	-	LncBook	antisense	Late LUAD
BPTF	HSALNG0118323	chr17	67889911	67893556	+	LncBook	sense	Late LUAD
BPTF	HSALNG0118325	chr17	67944958	67945197	+	LncBook	sense	Late LUAD
BPTF	HSALNG0118326	chr17	67950885	67951746	-	LncBook	antisense	Late LUAD
BPTF	HSALNG0145135	chr17	67952287	68019973	+	RefLnc	sense	Late LUAD
BPTF	HSALNG0118327	chr17	67955314	67957291	+	LncBook	sense	Late LUAD
STIM2	HSALNG0033503	chr4	26858288	26858953	+	LncBook	sense	Late LUAD
STIM2	HSALNG0033504	chr4	26859624	26860662	-	BestRefSeq	antisense	Late LUAD
STIM2	HSALNG0033505	chr4	26861722	26992923	-	LncBook	antisense	Late LUAD
STIM2	HSALNG0033510	chr4	27025314	27025765	-	LncBook	antisense	Late LUAD
BAIAP2	HSALNG0119188	chr17	80339898	81355859	-	LncBook	antisense	Late LUAD
BAIAP2	HSALNG0119225	chr17	81029988	81060973	+	LncBook	sense	Late LUAD
BAIAP2	HSALNG0119227	chr17	81110413	81113855	-	LncBook	antisense	Late LUAD
APOE	HSALNG0126510	chr19	44908869	44910640	-	LncBook	antisense	Late LUAD
APOE	HSALNG0126511	chr19	44909375	44919349	+	HAVANA	sense	Late LUAD
CALCRL	HSALNG0020989	chr2	187003220	187558051	+	LncBook	antisense	Late LUSC
CALCRL	HSALNG0020993	chr2	187342021	187343228	+	LncBook	antisense	Late LUSC

Gene	lncRNA	Chr	Start	End	S	DB	Type	Phenotype
KDM2A	HSALNG0085103	chr11	67074415	67148263	+	LncBook	sense	Late LUSC
KDM2A	HSALNG0143005	chr11	67123304	67124431	+	FANTOM	sense	Late LUSC
KDM2A	HSALNG0085111	chr11	67154732	67156889	+	LncBook	sense	Late LUSC
KDM2A	HSALNG0085112	chr11	67185879	67186130	+	LncBook	sense	Late LUSC
KDM2A	HSALNG0085113	chr11	67188158	67188667	+	LncBook	sense	Late LUSC
KDM2A	HSALNG0143205	chr11	67252336	67261545	-	HAVANA	antisense	Late LUSC
KDM2A	HSALNG0085117	chr11	67256131	67258341	-	LncBook	antisense	Late LUSC

Supplementary Table S3

Dark biomarkers of the late-stage lung cancers. The dark biomarkers were detected in LUAD and LUSC (denoted in the column “Subtype”), respectively. The columns “ID” and “Gene” gave the ENSEMBL ID and gene symbol of each detected dark biomarker. The columns “mqTrans-p” and “raw-p” gave the statistical t-test p-values of the engineered mqTrans values and the original expression levels of each dark biomarker in the late-stage lung cancers compared with the early-stage lung cancers of the same subtype. The chromosome, start position, end position, and strand of each dark biomarker were listed in the last five columns “Chr”, “Start”, “End”, and “S”.

Subtype	ID	Gene	mqTrans-p	raw-p	Chr	Start	End	S
LUAD	ENSG00000267249	RP11-973H7.3	8.28E-04	0.4012	chr18	12670426	12671145	-
LUAD	ENSG00000162836	ACP6	1.24E-03	0.0638	chr1	147629652	147670524	-
LUAD	ENSG00000072121	ZFYVE26	2.02E-03	0.0617	chr14	67727374	67816590	-
LUAD	ENSG00000141560	FN3KRP	3.20E-03	0.2761	chr17	82716706	82730328	+
LUAD	ENSG00000164603	C7orf60	3.95E-03	0.4486	chr7	112819147	112939875	-
LUAD	ENSG00000138030	KHK	5.22E-03	0.1535	chr2	27086747	27100762	+
LUAD	ENSG00000075945	KIFAP3	5.22E-03	0.2720	chr1	169921326	170085208	-
LUAD	ENSG00000105520	PLPPR2	5.94E-03	0.1081	chr19	11355386	11365698	+
LUAD	ENSG00000116353	MECR	1.12E-02	0.0730	chr1	29167696	29230942	-
LUAD	ENSG00000171634	BPTF	1.83E-02	0.0973	chr17	67825503	67984378	+
LUAD	ENSG00000109689	STIM2	2.23E-02	0.0926	chr4	26857601	27025381	+
LUAD	ENSG00000134072	CAMK1	2.37E-02	0.7634	chr3	9757347	9769992	-
LUAD	ENSG00000175866	BAIAP2	3.91E-02	0.1469	chr17	81035122	81117434	+
LUAD	ENSG00000130203	APOE	4.80E-02	0.4755	chr19	44905791	44909393	+

LUSC	ENSG00000064989	CALCRL	2.34E-02	0.0651	chr2	187341964	187448460	-
LUSC	ENSG00000173120	KDM2A	3.22E-02	0.7282	chr11	67119263	67258082	+

Supplementary Table S4

Dark biomarkers of the GSE190725 endocrine cells and endocrine progenitor. The "bulk" and "sc" stand for bulk RNA-seq dataset and scRNA-seq dataset, "overlap " represents the same dark biomarker in both datasets, where 1 means it is an overlap gene and 0 is not.

GSE190725 endocrine cells		overlap	GSE190725 endocrine progenitor		overlap
bulk	sc		**bulk**	**sc**	
1255_g_at	1320_at	0	1431_at	1552257_a_at	1
1552261_at	1431_at	0	1552256_a_at	1552261_at	1
1552266_at	1494_f_at	0	1552257_a_at	1552264_a_at	0
1552274_at	1552263_at	0	1552261_at	1552278_a_at	0
1552289_a_at	1552269_at	0	1552271_at	1552281_at	0
1552293_at	1552277_a_at	0	1552275_s_at	1552283_s_at	0
1552302_at	1552306_at	0	1552276_a_at	1552301_a_at	1
1552304_at	1552307_a_at	0	1552280_at	1552303_a_at	0
1552310_at	1552315_at	0	1552286_at	1552309_a_at	0

1552314_a_at	1552318_at	0	1552301_a_at	1552314_a_at	0
1552329_at	1552319_a_at	0	1552304_at	1552315_at	0
1552332_at	1552323_s_at	0	1552310_at	1552316_a_at	0
1552334_at	1552344_s_at	1	1552319_a_at	1552319_a_at	1
1552344_s_at	1552348_at	0	1552332_at	1552320_a_at	0
1552355_s_at	1552370_at	1	1552344_s_at	1552326_a_at	0
1552360_a_at	1552373_s_at	1	1552347_at	1552329_at	0
1552362_a_at	1552399_a_at	0	1552349_a_at	1552343_s_at	0
1552370_at	1552412_a_at	0	1552355_s_at	1552347_at	1
1552372_at	1552438_a_at	0	1552367_a_at	1552348_at	0
1552373_s_at	1552440_at	1	1552373_s_at	1552362_a_at	0
1552396_at	1552448_a_at	0	1552379_at	1552377_s_at	0
1552398_a_at	1552491_at	0	1552386_at	1552381_at	0
1552415_a_at	1552493_s_at	0	1552394_a_at	1552383_at	0
1552421_a_at	1552494_at	0	1552395_at	1552390_a_at	0
1552440_at	1552501_a_at	0	1552409_a_at	1552410_at	0
1552449_a_at	1552502_s_at	0	1552412_a_at	1552412_a_at	1
1552450_a_at	1552508_at	0	1552418_at	1552424_at	0
1552458_at	1552518_s_at	0	1552440_at	1552432_at	0

1552459_a_at	1552523_a_at	0	1552449_a_at	1552450_a_at	0
1552463_at	1552538_a_at	0	1552453_a_at	1552461_at	0
1552468_a_at	1552557_a_at	0	1552457_a_at	1552466_x_at	0
1552474_a_at			1552458_at	1552468_a_at	0
1552489_s_at			1552459_a_at	1552486_s_at	0
1552510_at			1552470_a_at	1552504_a_at	0
1552514_at			1552472_a_at	1552511_a_at	0
1552521_a_at			1552473_at	1552528_at	0
1552524_at			1552482_at	1552532_a_at	0
1552546_a_at			1552485_at	1552540_s_at	1
1552563_a_at			1552501_a_at	1552544_at	0
1552566_at			1552506_at	1552557_a_at	0
			1552514_at		
			1552518_s_at		
			1552535_at		
			1552540_s_at		
			1552548_at		

Supplementary Figure S1

Distributions of the t-test p-values of the original expression levels and the mqTrans values of the 13 dark biomarkers. The vertical axis was the "-log10()" scale of the t-test p-values, while the horizontal axis listed the 13 dark biomarkers. Some bins of the weak dark biomarkers may show smaller heights due to that they were not differentially expressed there. A higher bar suggested a more significant difference between the lung cancer and control groups. Some bars were not recognizable due to that they didn't show differential expressions. The horizontal line was plotted for -log10(0.05) as a baseline boundary.

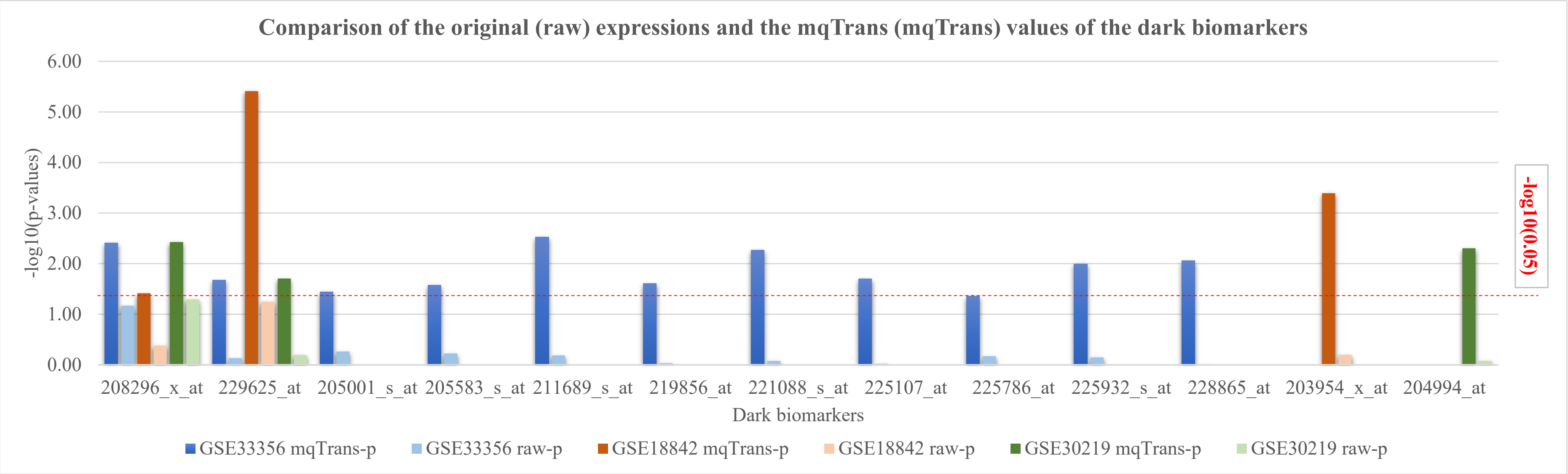

Supplementary Figure S2

Expression profiles of the two strong dark biomarkers GBP5 and TNFAIP8. Gene expression profiles of (a) GBP5 and (b) TNFAIP8 across different organs were retrieved from the database GTEx Portal. The gene expression profiles of (c) GBP5 and (d) TNFAIP8 across different cancer types compared against their matched normal samples were retrieved from the GEPIA database using the TCGA data. The body map and average expression levels of each strong dark biomarker across different cancer types and the matched normal samples were illustrated. The expression levels were in the log scale.

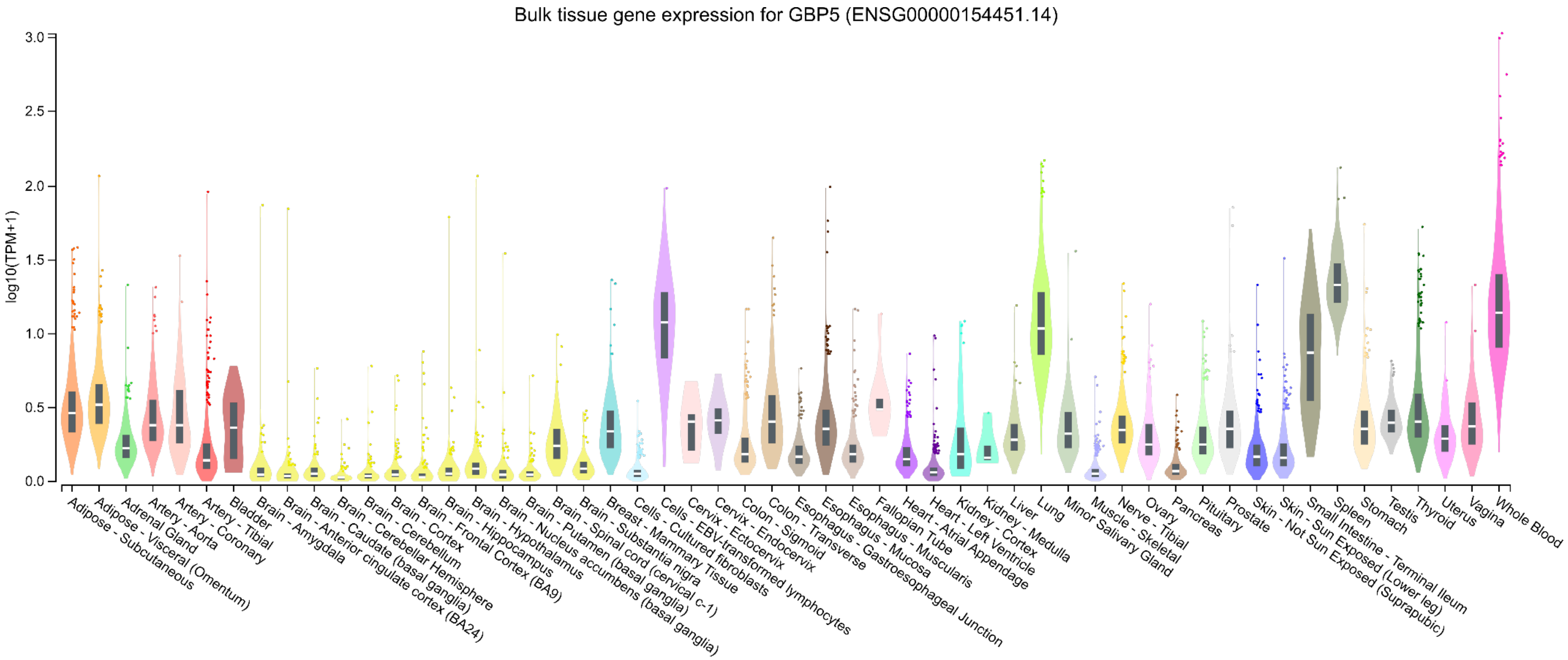
Bulk tissue gene expression for GBP5 (ENSG00000154451.14)
log10(TPM+1)
3.0
2.5
2.0
1.5
1.0
0.5
0.0
Adipose - Subcutaneous
Adipose - Visceral (Omentum)
Adrenal Gland
Artery - Aorta
Artery - Coronary
Artery - Tibial
Bladder
Brain - Amygdala
Brain - Anterior cingulate cortex (BA24)
Brain - Caudate (basal ganglia)
Brain - Cerebellar Hemisphere
Brain - Cerebellum
Brain - Cortex
Brain - Frontal Cortex (BA9)
Brain - Hippocampus
Brain - Hypothalamus
Brain - Nucleus accumbens (basal ganglia)
Brain - Putamen (basal ganglia)
Brain - Spinal cord (cervical c-1)
Brain - Substantia nigra
Breast - Mammary Tissue
Cells - Cultured fibroblasts
Cells - EBV-transformed lymphocytes
Cervix - Ectocervix
Cervix - Endocervix
Colon - Sigmoid
Colon - Transverse
Esophagus - Gastroesophageal Junction
Esophagus - Mucosa
Esophagus - Muscularis
Fallopian Tube
Heart - Atrial Appendage
Heart - Left Ventricle
Kidney - Cortex
Kidney - Medulla
Liver
Lung
Minor Salivary Gland
Muscle - Skeletal
Nerve - Tibial
Ovary
Pancreas
Pituitary
Prostate
Skin - Not Sun Exposed (Suprapubic)
Skin - Sun Exposed (Lower leg)
Small Intestine - Terminal Ileum
Spleen
Stomach
Testis
Thyroid
Uterus
Vagina
Whole Blood

(a)

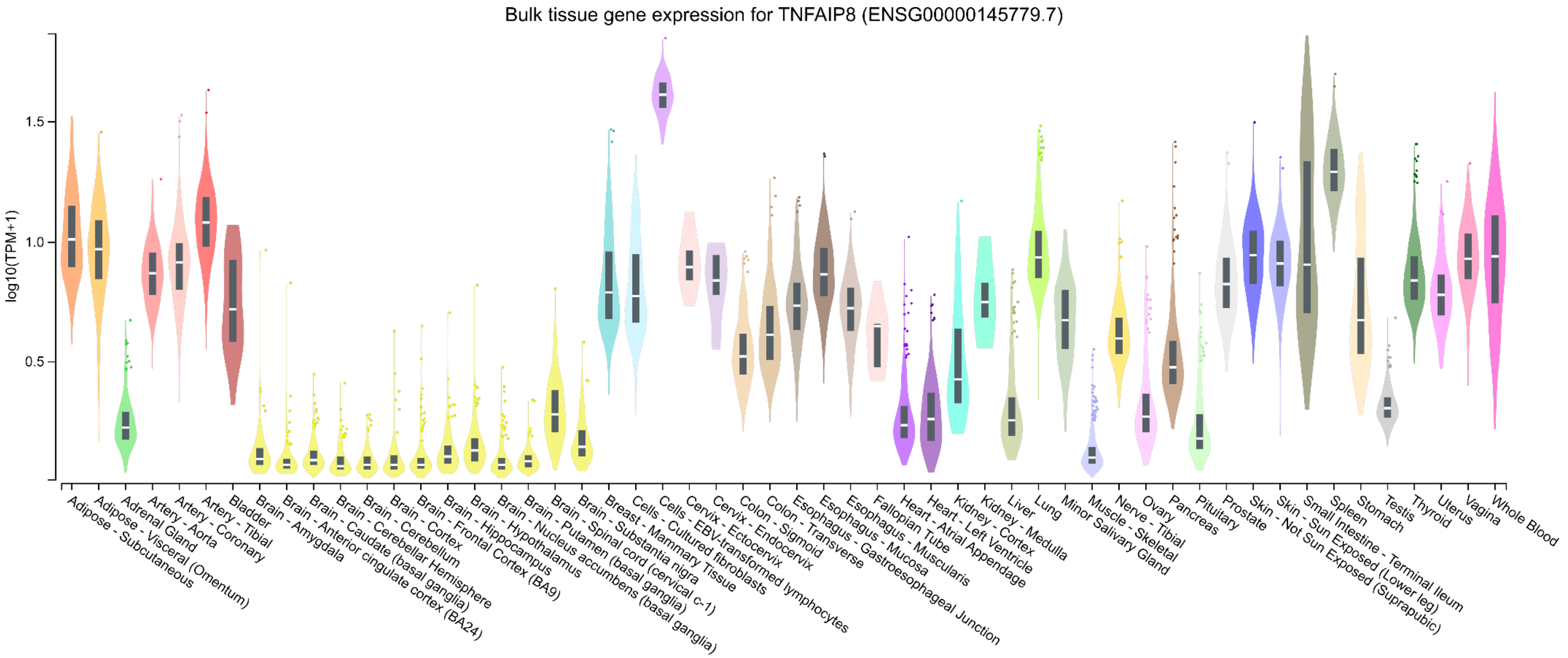

Bulk tissue gene expression for TNFAIP8 (ENSG00000145779.7)
log10(TPM+1)
1.5
1.0
0.5
Adipose - Subcutaneous
Adipose - Visceral (Omentum)
Adrenal Gland
Artery - Aorta
Artery - Coronary
Artery - Tibial
Bladder
Brain - Amygdala
Brain - Anterior cingulate cortex (BA24)
Brain - Caudate (basal ganglia)
Brain - Cerebellar Hemisphere
Brain - Cerebellum
Brain - Cortex
Brain - Frontal Cortex (BA9)
Brain - Hippocampus
Brain - Hypothalamus
Brain - Nucleus accumbens (basal ganglia)
Brain - Putamen (basal ganglia)
Brain - Spinal cord (cervical c-1)
Brain - Substantia nigra
Breast - Mammary Tissue
Cells - Cultured fibroblasts
Cells - EBV-transformed lymphocytes
Cervix - Ectocervix
Cervix - Endocervix
Colon - Sigmoid
Colon - Transverse
Esophagus - Gastroesophageal Junction
Esophagus - Mucosa
Esophagus - Muscularis
Fallopian Tube
Heart - Atrial Appendage
Heart - Left Ventricle
Kidney - Cortex
Kidney - Medulla
Liver
Lung
Minor Salivary Gland
Muscle - Skeletal
Nerve - Tibial
Ovary
Pancreas
Pituitary
Prostate
Skin - Not Sun Exposed (Suprapubic)
Skin - Sun Exposed (Lower leg)
Small Intestine - Terminal Ileum
Spleen
Stomach
Testis
Thyroid
Uterus
Vagina
Whole Blood

(b)

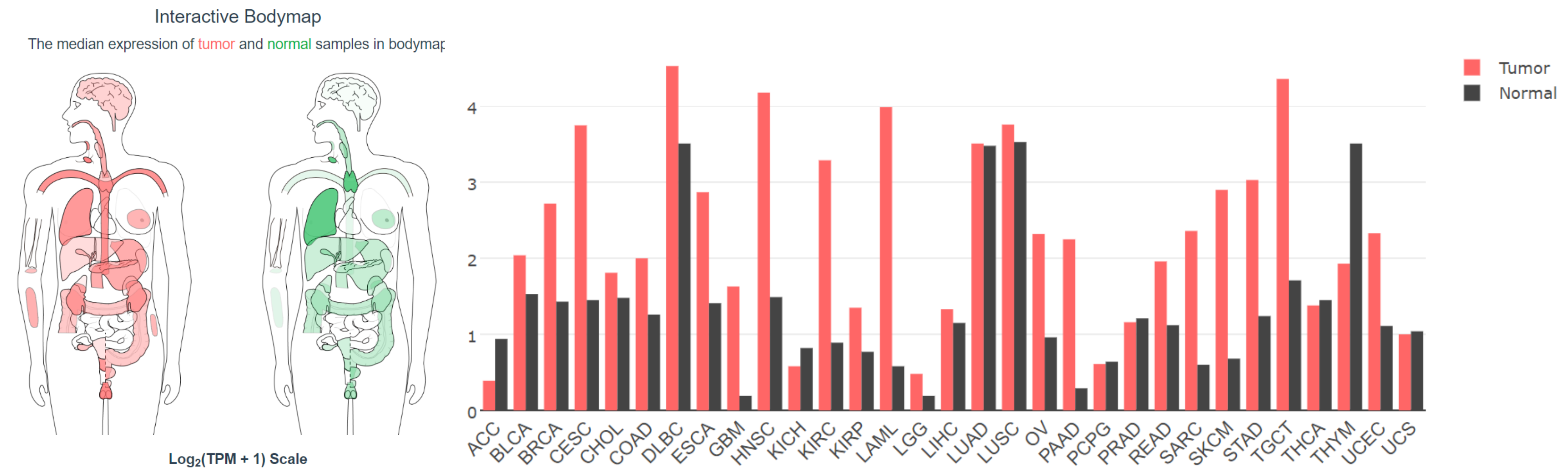
Interactive Bodymap
The median expression of tumor and normal samples in bodymap
Log2(TPM + 1) Scale
Tumor
Normal
0
1
2
3
4
ACC
BLCA
BRCA
CESC
CHOL
COAD
DLBC
ESCA
GBM
HNSC
KICH
KIRC
KIRP
LAML
LGG
LIHC
LUAD
LUSC
OV
PAAD
PCPG
PRAD
READ
SARC
SKCM
STAD
TGCT
THCA
THYM
UCEC
UCS

(c)

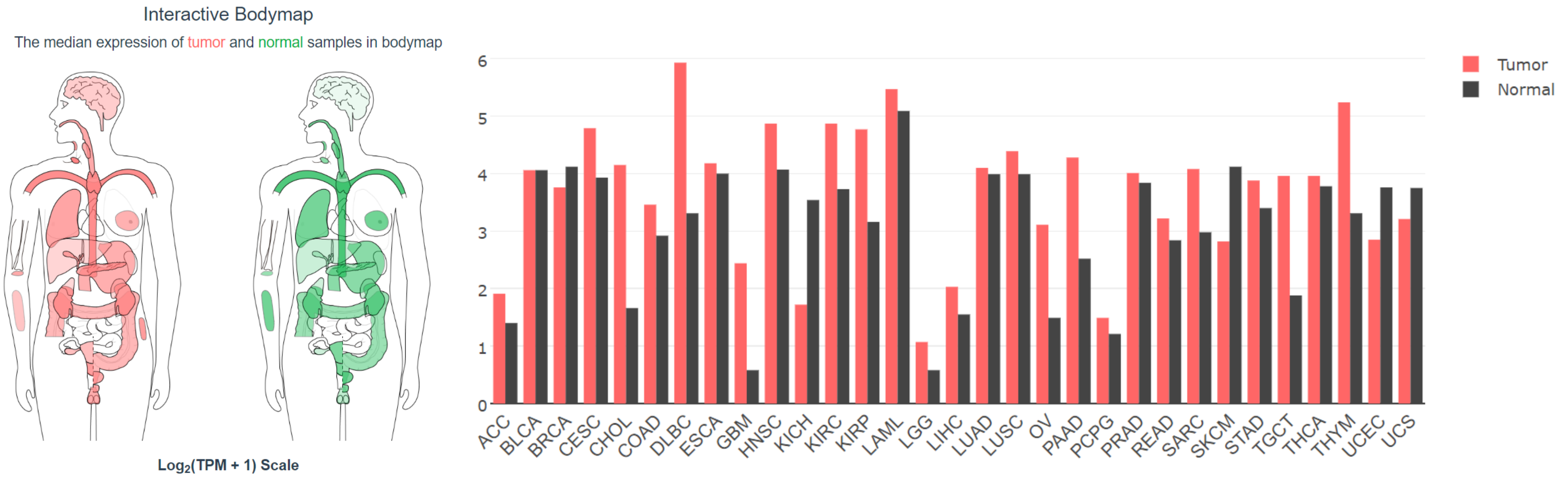
Interactive Bodymap
The median expression of tumor and normal samples in bodymap
Log2(TPM + 1) Scale
Tumor
Normal
0
1
2
3
4
5
6
ACC
BLCA
BRCA
CESC
CHOL
COAD
DLBC
ESCA
GBM
HNSC
KICH
KIRC
KIRP
LAML
LGG
LIHC
LUAD
LUSC
OV
PAAD
PCPG
PRAD
READ
SARC
SKCM
STAD
TGCT
THCA
THYM
UCEC
UCS

(d)

Supplementary Figure S3

Expression levels of the novel transcript ENSG00000267249 (gene symbol: RP11-973H7.3) across different organs. The data was retrieved from the UCSC Genome Browser [1].

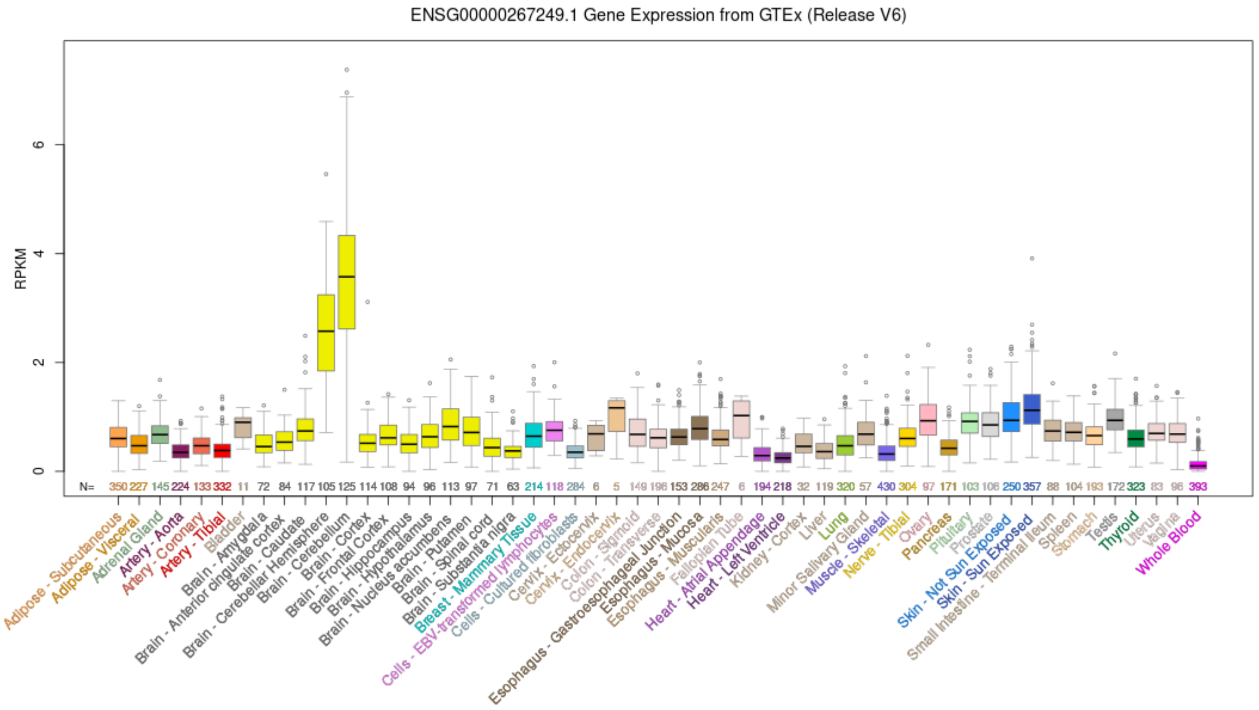

Supplementary Figure S4

Kaplan Meier (KM) survival analysis of the 16 dark biomarkers of the two lung cancer subtypes LUAD and LUSC. Each sub-figure gave the KM plots of the original expression levels and the mqTrans values of each dark biomarker on the left and right parts, respectively.

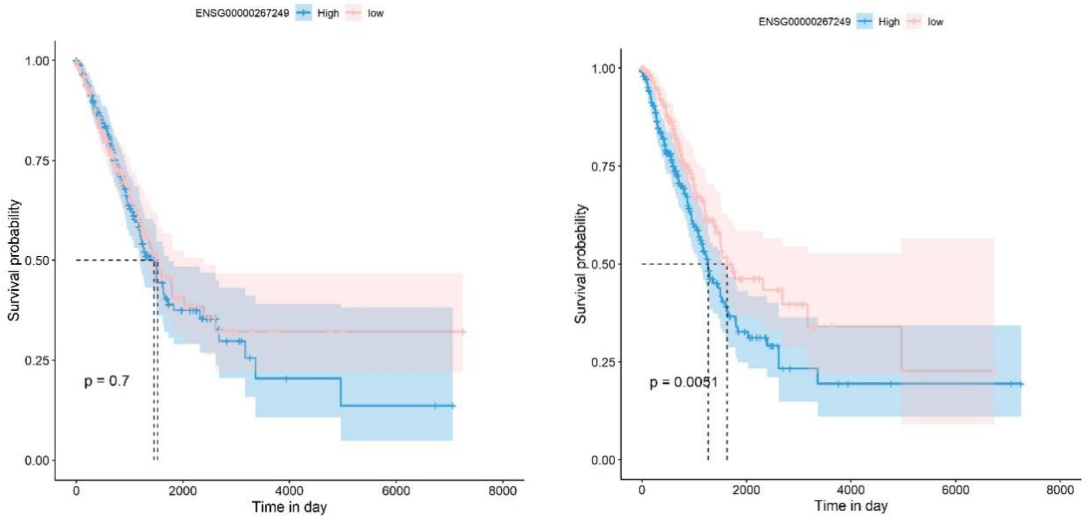

(a) ENSG00000267249 in LUAD

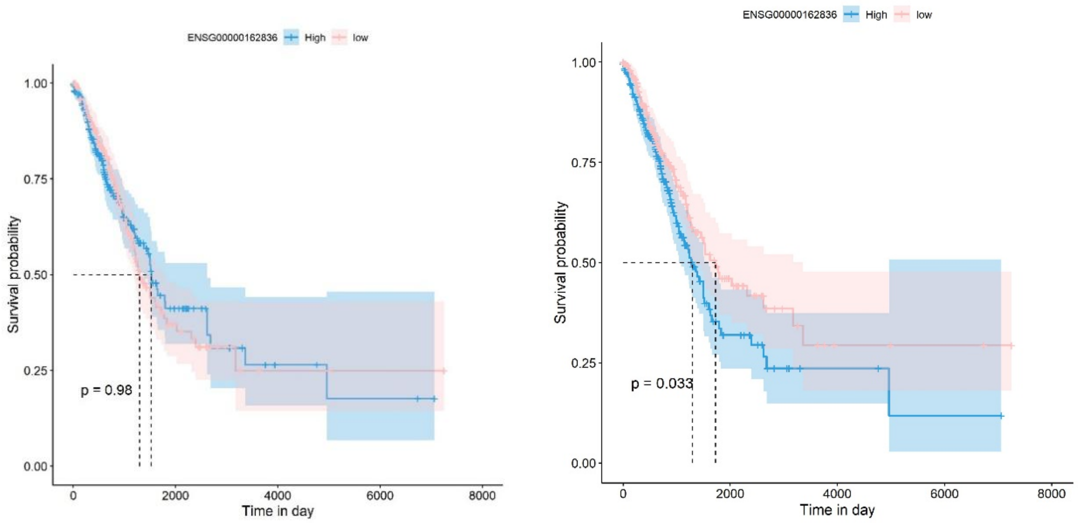

(b) ENSG00000162836 in LUAD

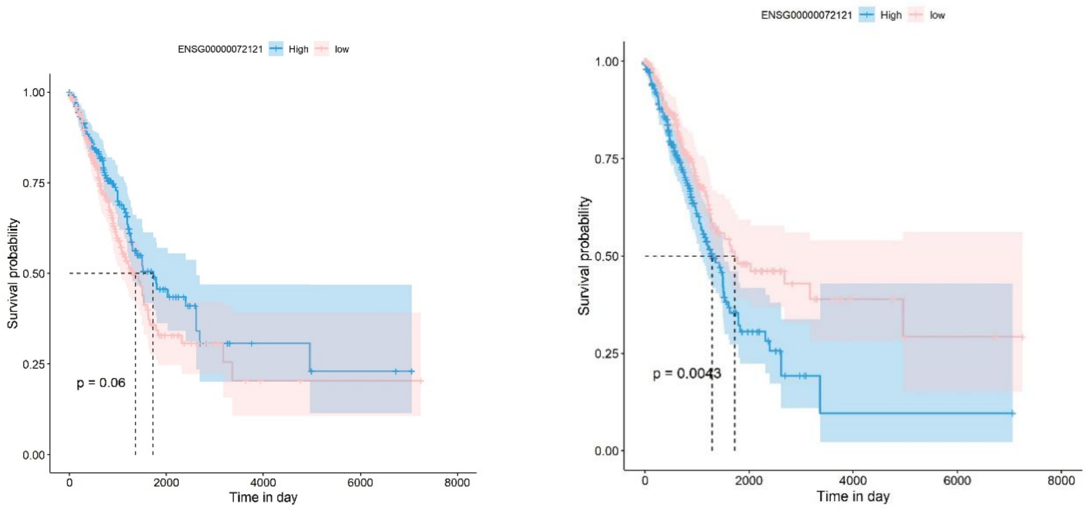

(c) ENSG00000072121 in LUAD

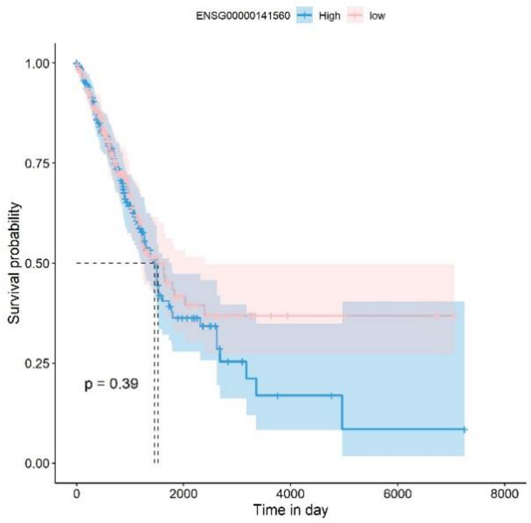

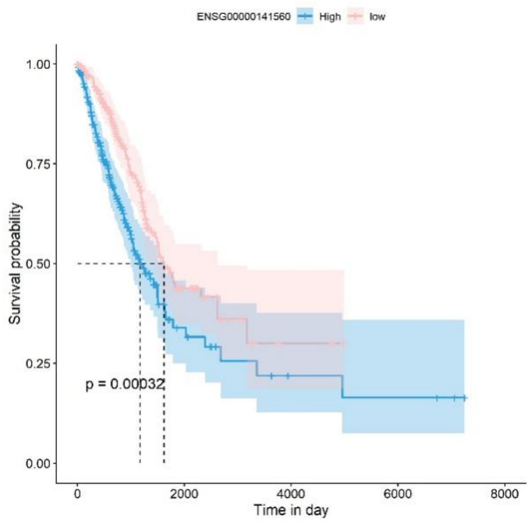

(d) ENSG00000141560 in LUAD: Original on the left, mqTrans on the right

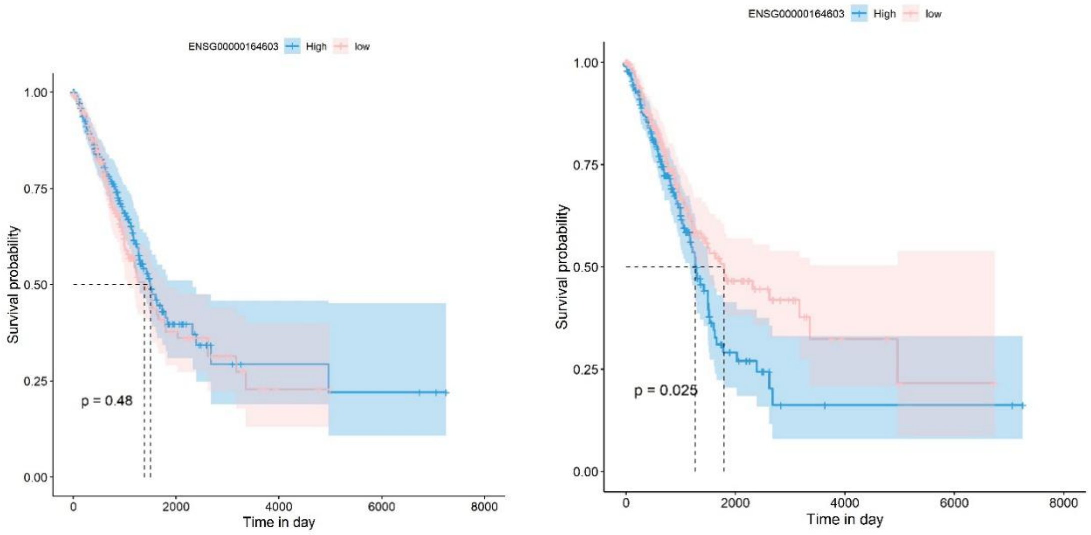

(e) ENSG00000164603 in LUAD

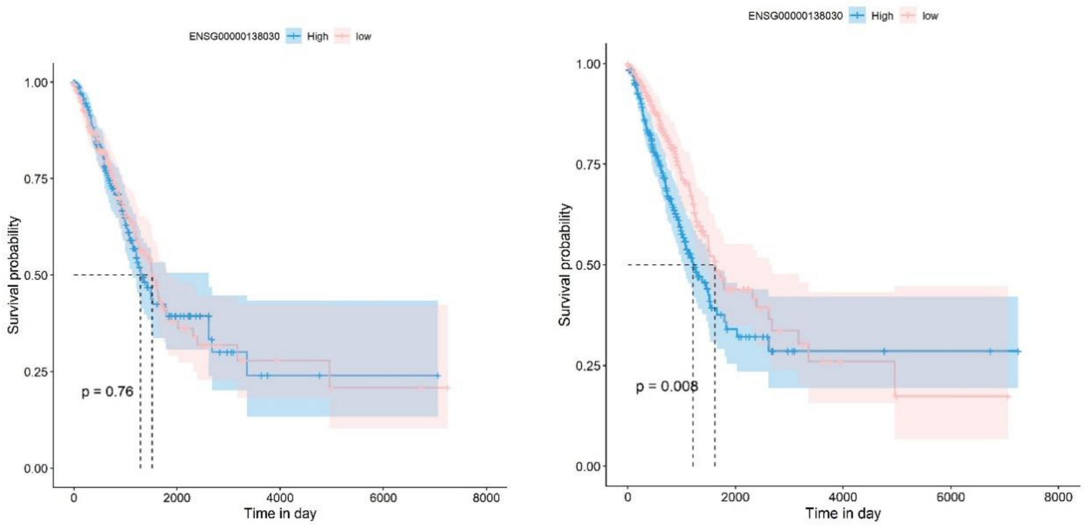

(f) ENSG00000138030 in LUAD

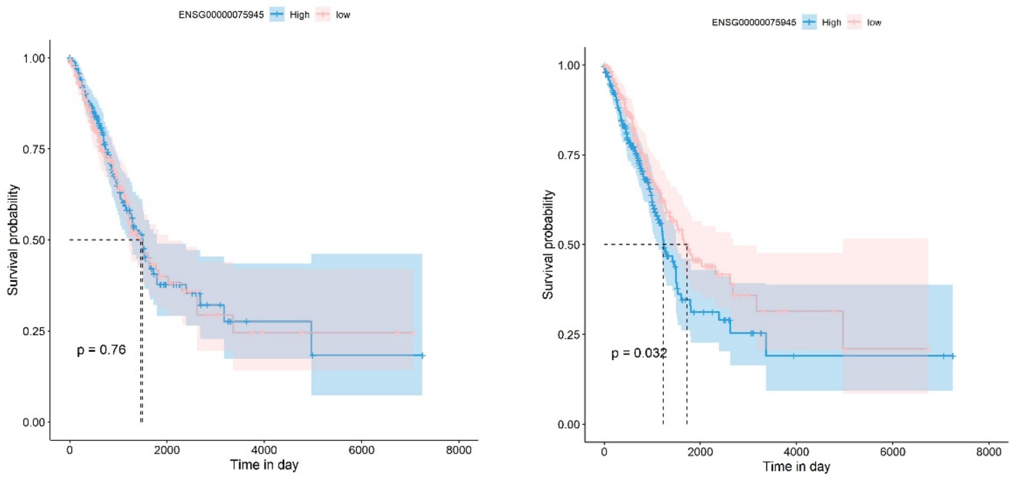

(g) ENSG00000075945 in LUAD

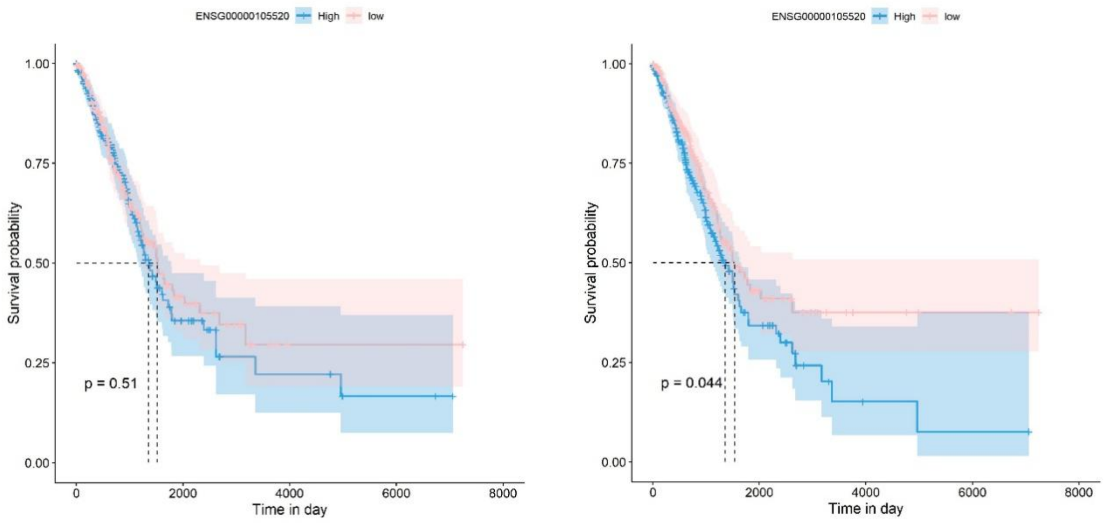

(h) ENSG00000105520 in LUAD

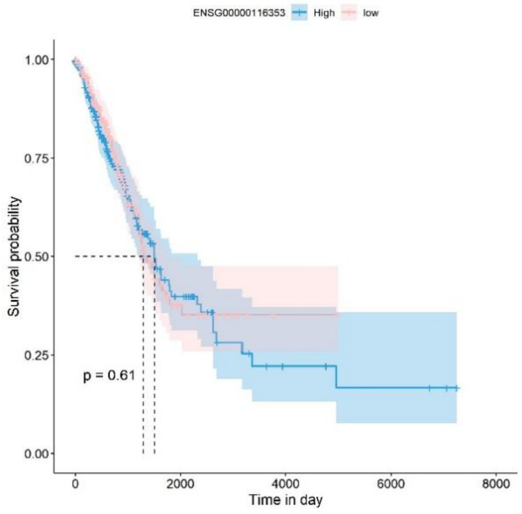

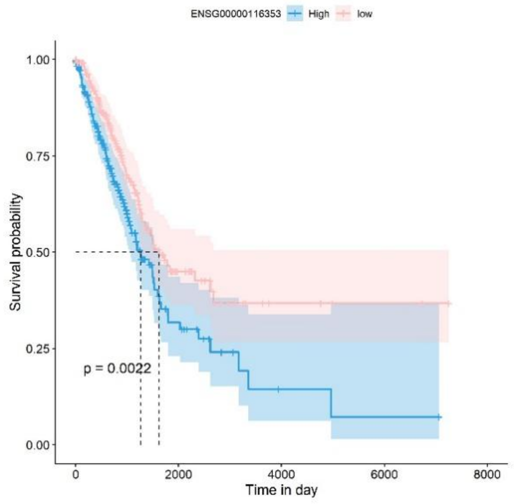

(i) ENSG00000116353 in LUAD

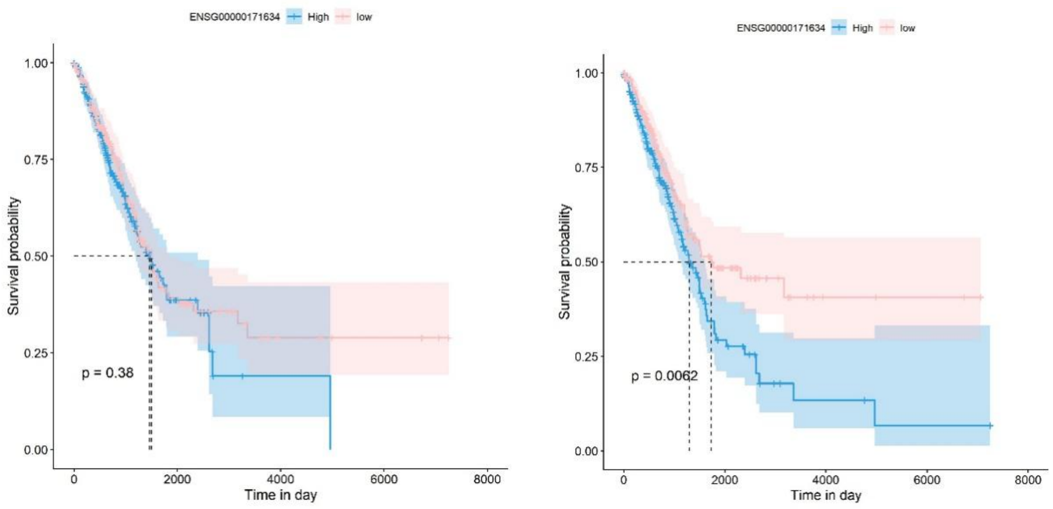

(j) ENSG00000171634 in LUAD

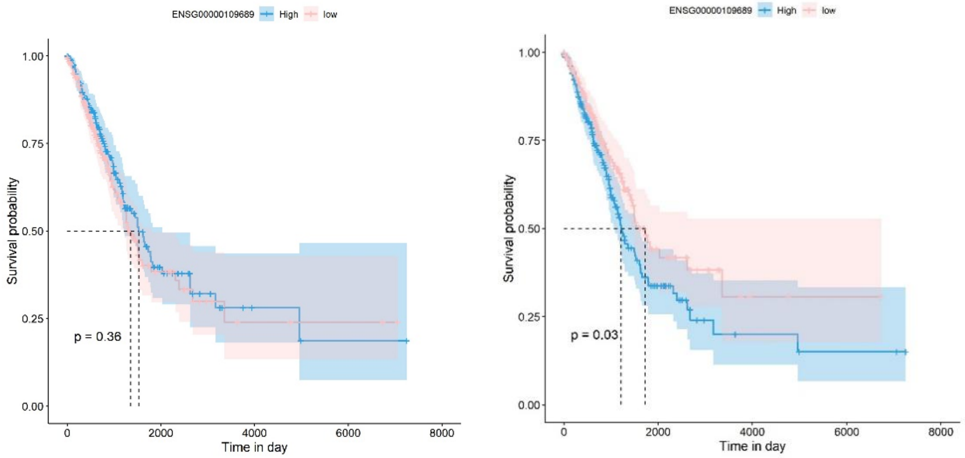

(k) ENSG00000109689 in LUAD

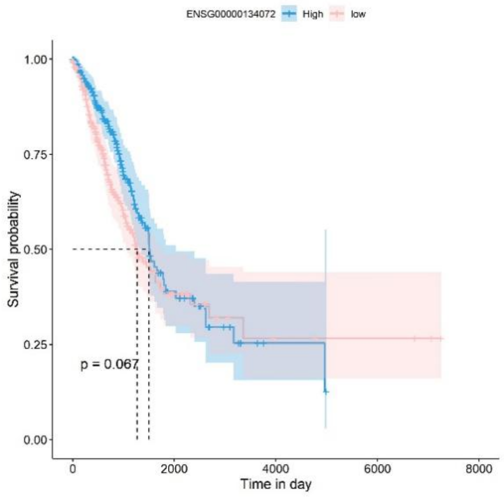

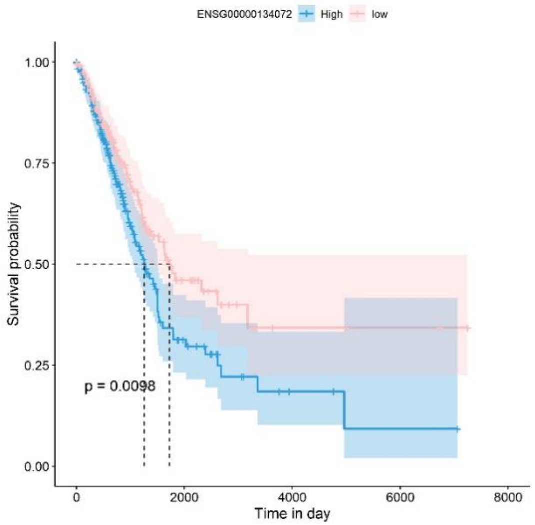

(l) ENSG00000134072 in LUAD

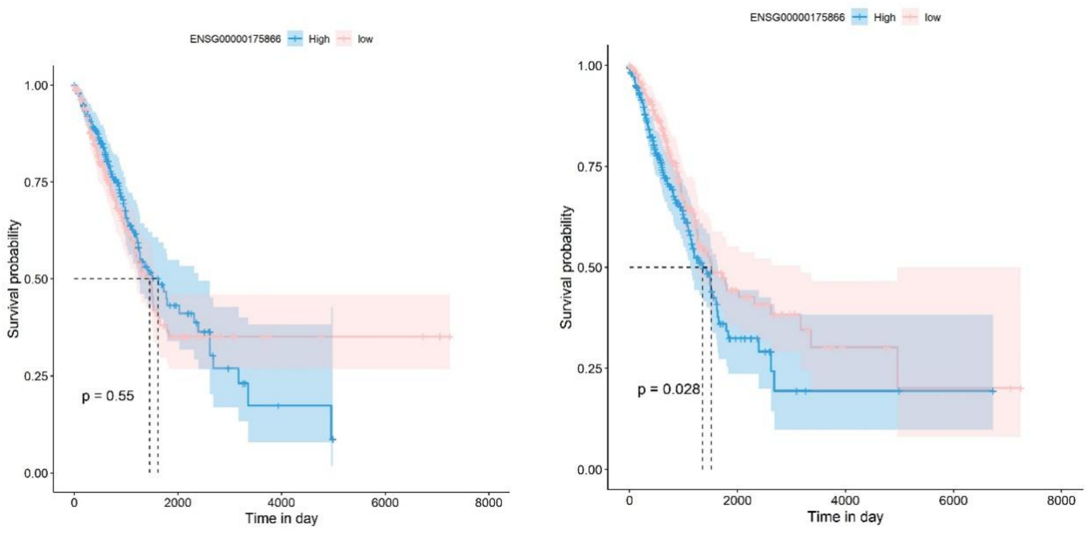

(m) ENSG00000175866 in LUAD

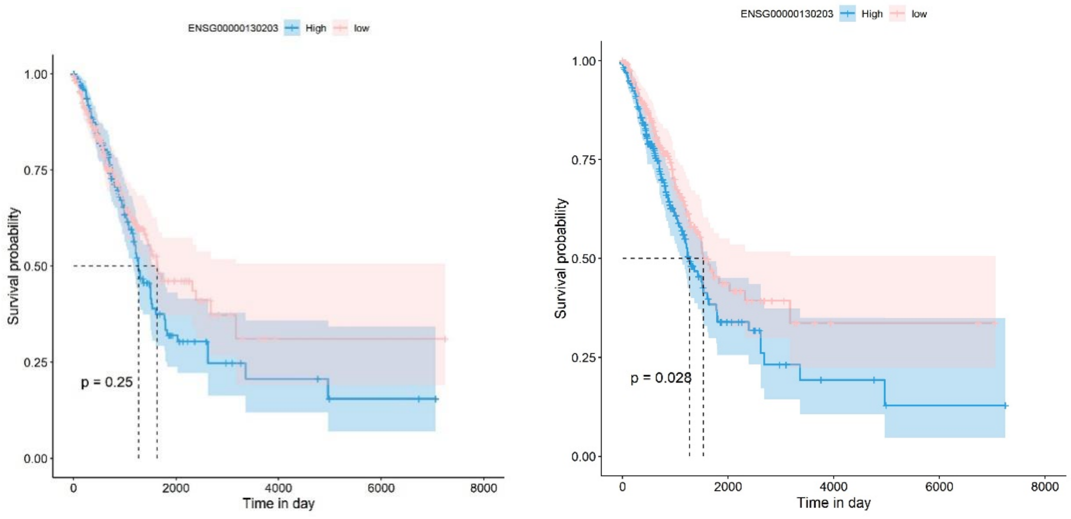

(n) ENSG00000130203 in LUAD

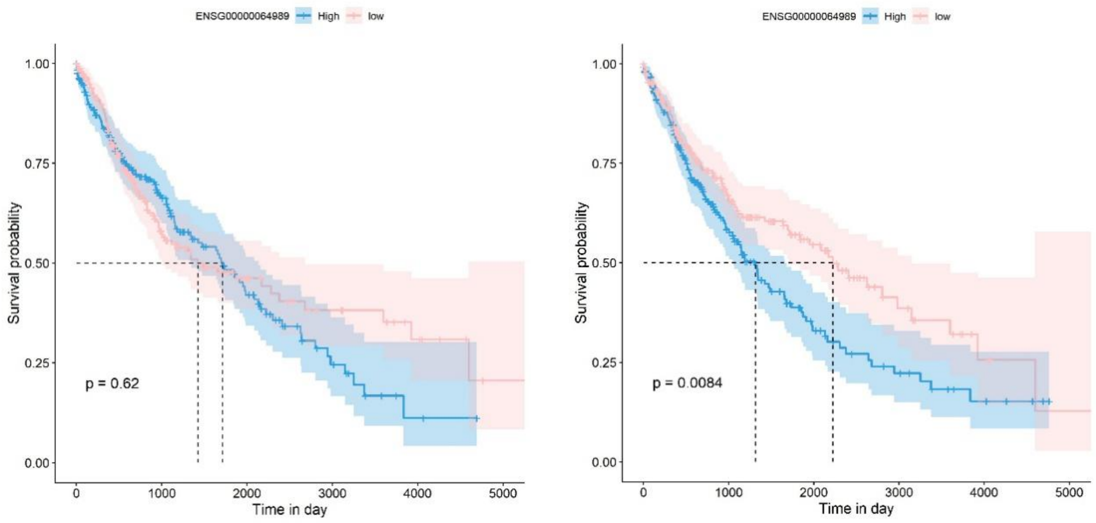

(o) ENSG00000064989 in LUSC

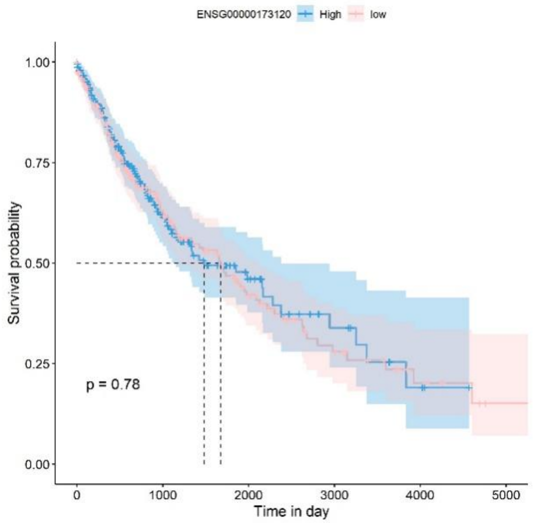

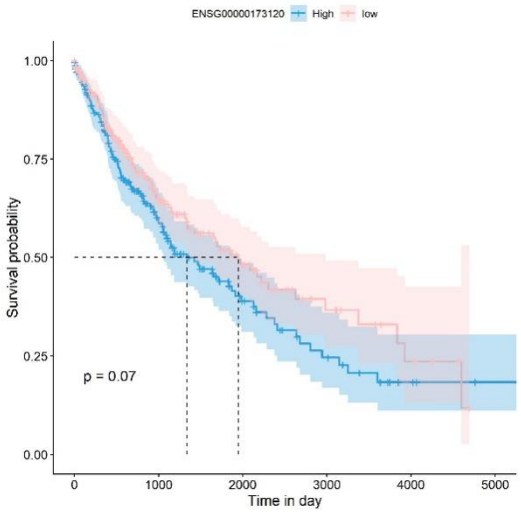

(p) ENSG00000173120 in LUSC

Supplementary Figure S5

Expression profiles of the two strong dark biomarkers (GBP5 and TNFAIP8) and two biomarker genes (CD200 and CD200R1) differentially expressed in lung cancer. The gene expression profiles of (a) GBP5, (b) TNFAIP8, (c) CD200 and (d) CD200R1 across different cancer types compared against their matched normal samples were retrieved from the GEPIA database using the TCGA data. The average expression levels of each gene across different cancer types and the matched normal samples were illustrated. The expression levels were in the log scale.

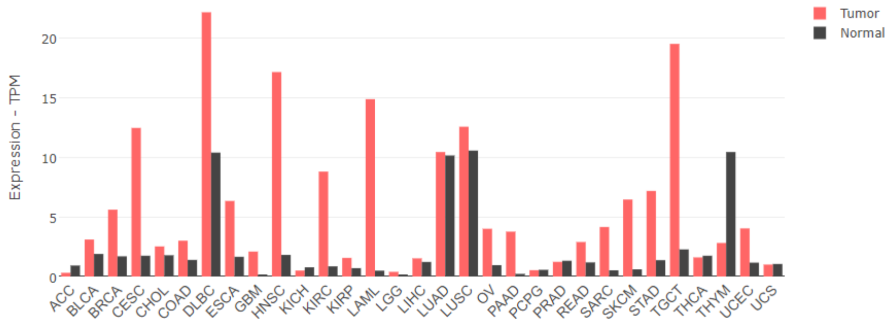

(a)

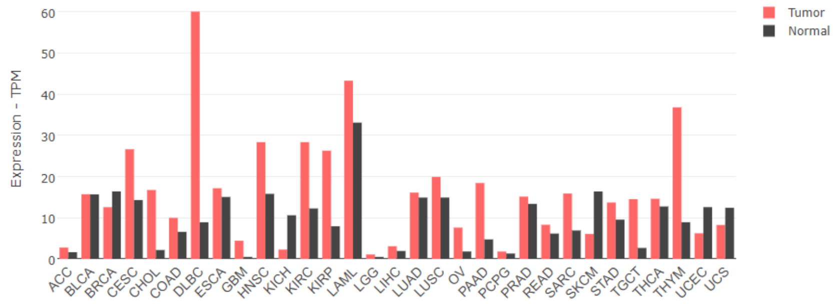

(b)

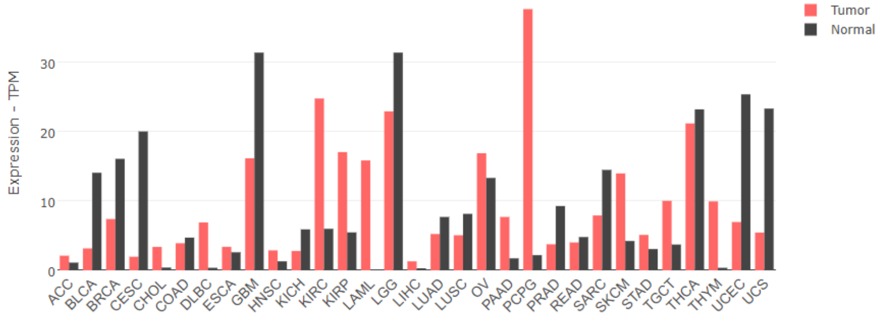

(c)

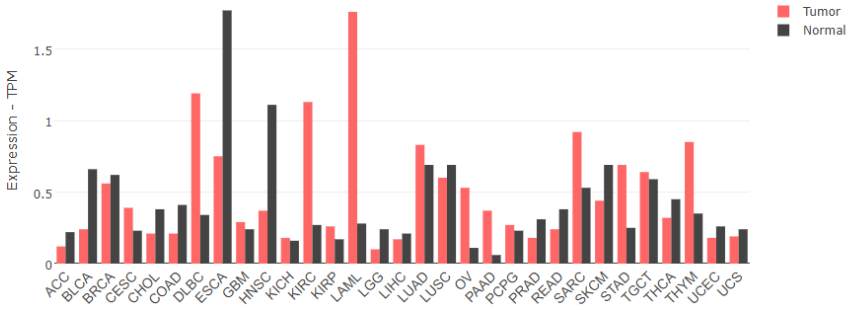

(d)

References

1. Kent, W.J.; Sugnet, C.W.; Furey, T.S.; Roskin, K.M.; Pringle, T.H.; Zahler, A.M.; Haussler, D. The human genome browser at UCSC. *Genome Res* **2002**, *12*, 996-1006, doi:10.1101/gr.229102.
